# Changes in Phytochemical Profiles and Biological Activity of Olive Leaves Treated by Two Drying Methods

**DOI:** 10.3389/fnut.2022.854680

**Published:** 2022-04-28

**Authors:** Chengcheng Zhang, Jianming Zhang, Xiaoting Xin, Shenlong Zhu, Erli Niu, Qinghang Wu, Ting Li, Daqun Liu

**Affiliations:** ^1^Food Science Institute, Zhejiang Academy of Agricultural Sciences, Hangzhou, China; ^2^Institute of Crop and Nuclear Technology Utilization, Zhejiang Academy of Agricultural Sciences, Hangzhou, China

**Keywords:** olive leaves, phenolic compounds, triterpenic acids, biological activities, drying

## Abstract

Olive leaves, which are the most abundant byproducts of the olive industry, offer multiple health benefits. The investigation of the phytochemical profiles and relevant biological activities is an essential step toward transforming these low-value byproducts into value-added ones. This study systematically investigated the phytochemical profiles, antioxidant capacity, and inhibition rates of olive leaves from four cultivars on the α-glucosidase, α-amylase, and angiotensin-converting enzyme (ACE). The leaves were prepared using two common drying methods, namely, hot air-drying and freeze-drying. A total of 33 bioactive compounds were identified in the olive leaves, namely, 19 flavonoids, 2 phenylethanoids, 2 coumarins, 2 hydroxycinnamic acids, 2 iridoids, and 6 triterpenic acids. Quantification of the bioactive compounds revealed high amounts of polyphenols, especially flavonoids [2,027–8,055 mg/kg dry weight (DW)], iridoids (566–22,096 mg/kg DW), and triterpenic acids (13,824–19,056 mg/kg DW) in the olive leaves. The hot air-dried leaves showed significantly (*P* < 0.05) higher iridoid (oleuropein and secoxyloganin) content than the fresh leaves, while freeze-drying resulted in significantly (*P* < 0.05) higher flavonoid aglycone and hydroxytyrosol content. Additionally, freeze-drying led to samples with the highest radical scavenging, α-amylase, α-glucosidase, and ACE inhibition abilities. The flavonoid (e.g., quercetin, luteolin, eriodictyol, kaempferol-7-*O*-glucoside, and luteolin-7-*O*-glucoside), hydroxytyrosol, and oleanolic acid contents in the olive leaves were positively correlated (*P* < 0.05) with their bioactive potentials.

## Introduction

Olive (*Olea europaea* L.) leaves are byproducts generated from olive tree cultivation during tree pruning, fruits harvesting, and olive oil processing (1). Over one million tons of olive leaves are accumulated annually (2, 3); however, most olive leaves are burnt or discarded as waste, resulting in environmental pollution and loss of a potential resource. In recent decades, the comprehensive use of olive leaves has attracted much research interest, mainly due to their high content of valuable bioactive compounds, such as phenolic derivatives, phytosterols, tocols, and pentacyclic triterpenes (4). Olive leaf extracts have been used as natural antioxidants in meat products, olive oil, sunflower oil, and soybean oil (5, 6), incorporated as supplements in functional foods (1), and used to delay the microbiological spoilage of seafood (7). In addition, the reutilization of olive leaves to produce extracts rich in bioactive phytochemicals for use in pharmaceutical, nutraceutical, and food industries has shown to not only reduce the environmental burden but also add economic value (1, 4).

Olive leaves are also valuable due to their great biological potential. Indeed, the health benefits of olive leaves have been known since ancient times. Historically, this plant was widely used as a folk medicine for treating fever and other diseases, such as diabetes mellitus, hypertension, rheumatism, arrhythmia, and cancer (8). Today, several *in vitro* and *in vivo* investigations have endorsed the wide spectrum of biological properties of olive leaf extracts, including their antioxidant, antiviral, anti-fungal, anti-inflammatory, antimicrobial, and anti-carcinogenic activities (1, 9–11). These health-promoting properties of olive leaves have been mainly related to their phenolic content, as phenolic compounds possess strong antioxidant activity that protects against chronic diseases, such as type-2 diabetes, cardiovascular diseases, and inflammation (4). The main phenolic compositions of olive leaves were found to be secoiridoids, flavonoids, and simple phenols, such as oleuropein, luteolin, luteolin-7-*O*-glucoside, and hydroxytyrosol (12, 13), with phenolic compounds, the major bioactive phytochemicals in olive leaves. For example, oleuropein, the most abundant olive phenolic compound, was found to exhibit anti-inflammatory, antioxidant, and antimicrobial properties (14). Flavonoids, another major class of phenolics, have shown an ability to mitigate type 2 diabetes by inhibiting the activities of α-amylase and α-glucosidase (15). Moreover, hydroxytyrosol was found to possess a very strong antioxidant capacity through hydrogen donation and the scavenging of free radicals (16).

The phenolic profiles and biological activity of olive leaves may be affected by multiple factors, including genetics (cultivar/genotype), growing conditions (maturity, climate, and soil properties), and post-harvest processing (processing, preservation methods, and drying conditions) (12, 17–19). In particular, post-harvest processing such as drying has been reported to cause significant modification in the chemical compositions of the olive leaves and their antioxidant properties (12, 20). Drying is an indispensable technique for processing and preserving plant materials. Among the various drying methods, hot air-drying (HD) has been the most commonly employed technique for plant material preservation on an industrial scale; however, it usually causes the loss of bioactive compounds due to high temperatures (21, 22). Freeze drying (FD) has shown to be an effective drying method to retain nutrients, but it is limited by its long drying time and expensive costs (23). Several studies have also been conducted on phenolic variation of olive leaves under FD and HD, and the extracts obtained from hot air-dried (105°C) olive leaves showed increased recovery of total phenolic (twofold) and total flavonoid (threefold) content compared to fresh leaves (12). Moreover, Hussam et al. reported that the antioxidant properties of hot air-dried (120°C) olive leaves were significantly (*P* < 0.05) higher than the freeze-dried samples (20). However, until now, there has not been a comprehensive investigation of the changes of phytochemical profiles of olive leaves affected by FD and HD. In addition, the effects of the drying process on the biological potential of olive leaves are still unknown.

To optimize the utilization of olive leaves, the aim of this study was to compare the effects of FD and HD on the phytochemical profiles and biological activities of olive leaves. Specifically, this study: (i) comprehensively characterized the phytochemical profiles of olive leaves from four cultivars treated by FD and HD; (ii) systematically explored the variation in the *in vitro* biological activities (i.e., antioxidant, α-glucosidase, α-amylase, and ACE inhibition activities) of olive leaves affected by FD and HD; and (iii) further revealed correlations between the individual phytochemicals and overall biological activities of olive leaves. This work aims to provide theoretical guidance for producing value-added products from olive leaves.

## Materials and Methods

### Chemicals and Reagents

Folin-Ciocalteu’s phenol reagent (F9252, 2N), 2,4,6-tris (2-pyridyl)-s-triazine (TPTZ, ≥ 99%), 2,2-azino-bis (3-ethylbenzothiazoline-6-sulfonic acid) diammonium salt (ABTS, ≥ 99%), 2,2-diphenyl-1-picrylhydrazyl (DPPH, D9132), Trolox (≥ 97%), hippuryl-histidyl-leucine (HHL, ≥ 98%), *Saccharomyces cerevisiae* α-glucosidase (G0660, 28 units/mg solid), type VI-B porcine pancreatic α-amylase (A3176, 14 units/mg solid), and rabbit lung angiotensin-converting enzyme (A6778, ≥ 2.0 units/mg protein) were purchased from Sigma-Aldrich (St. Louis, MO, United States). Analytical standards (greater than 98%) of hydroxytyrosol, esculin, corosolic acid, ursolic acid, maslinic acid, oleanolic acid, taxifolin, luteolin, quercetin, kaempferol, apigenin, chlorogenic acid, plantamajoside, rutin, eriodictyol, tiliroside, apigenin-7-*O*-neohesperidoside, luteolin-7-*O*-glucoside, oleuropein, secoxyloganin, and gallic acid were purchased from Yuanye Bio-Technology Co., Ltd., (Shanghai, China). For chromatographic analysis, high-performance liquid chromatography (HPLC)-grade formic acid, acetic acid, and acetonitrile were obtained from Alfa Aesar (Shanghai, China) and Merck (Darmstadt, Germany). Ultrapure water was obtained from a Milli-Q system (Millipore, Bedford, MA, United States).

### Plant Materials and Drying Process

Olive leaves were collected from four cultivars. Two cultivars originated from Italy (I79 and Canino), one autochthonous cultivar was from China (Huaou5), and one cultivar was from Spain (Nevadillo fino). All of the olive leaf samples were randomly taken from at least three trees of the same cultivar between mid-November and mid-December, 2020. All of the cultivars were planted in the research garden of the Institute of Crops and Nuclear Technology Utilization at the Zhejiang Academy of Agricultural Sciences, China, under the same agronomic and environmental conditions. Table 1 summarizes the details of the olive leaves that were used in this study, as well as their pictures. After the fresh olive leaves were picked, they were randomly divided into three sub-groups.

**TABLE 1:** Four cultivars of olive leaves used in this study.

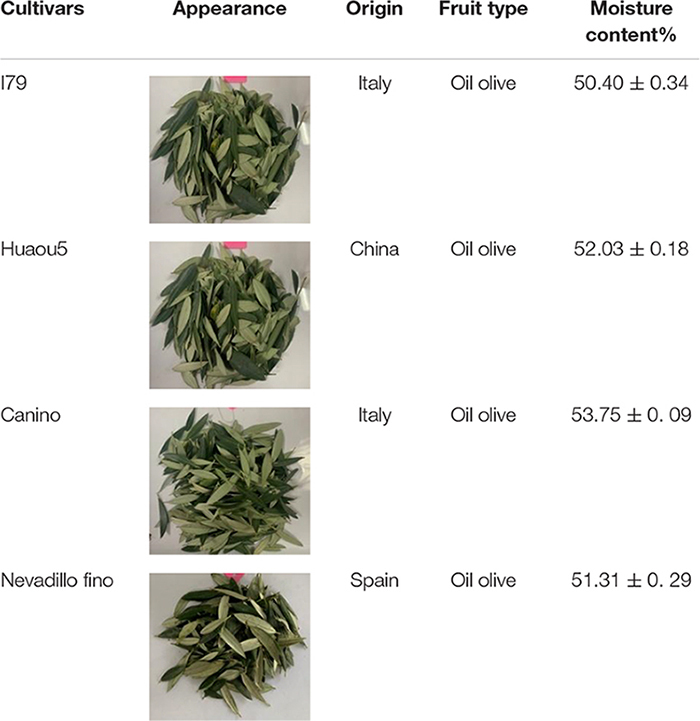

i) The fresh sub-group contained olive leaves from each cultivar (200 g without drying), which were stored in vacuum-sealed bags at −80°C.
ii) The hot air-dried sub-group contained fresh olive leaves that were dried in a hot air oven at 105°C (Jinghong DHG-9070A, Shanghai, China) for 90 min.
iii) The freeze-dried sub-group contained fresh olive leaves that were pre-frozen at −80°C for 12 h and then dried in a lyophilizer (Scientz SCIENTZ-18N, Ningbo, China) for 48 h under a vacuum pressure of 0.002 kPa. The temperatures within the cold trap and drying chamber were −55°C and −25°C, respectively.

Subsequently, all of the dried olive leaves were ground for 1 min at high speed in a micronizer (Baijie BJ-200, Hangzhou, China), sieved through a 100-mesh screen, and then stored at −20°C until extraction.

### Sample Extraction

Fresh olive leaves were homogenized using a breaking pulper (Solis-161 type, Guangzhou, China) for 10 min. Then, either dried olive leaf powder (1 g) or fresh homogenized samples (2 g) were added to 10 mL of 70% ethanol solution and extracted under continuous sonication (40 kHz) at 50°C for 30 min using a sonicator (KQ-5200DE, Kunshan Ultrasonic Instrument Co., Ltd., China). After extraction, the samples were centrifuged at 5,000 rpm for 10 min, and the supernatant and residue were collected, respectively. Afterward, 10 mL of 70% ethanol solution was added to the residue, and extraction was repeated as described above. After centrifugation, both supernatants were collected and mixed, diluted to 25 mL, and then stored at −20°C for further analysis.

### Chemical Profiling *via* UHPLC-Q-Exactive Orbitrap-MS

The phytochemical constituents were identified by an Ultimate 3000 UPLC system coupled with a Q-Exactive Orbitrap-MS spectrometer (Thermo Fisher Scientific, San Jose, CA, United States). Chromatographic analysis was performed using a Zorbax Eclipse C18 (2.1 × 100 mm^2^, 1.8 μm) column, with 0.1% formic acid in deionized water and acetonitrile as mobile phases A and B, respectively. A gradient was generated under the following conditions: 0–2 min, 5% B; 2–5 min, 5%–30% B; 6–7 min, 30% B; 7–12 min, 30%–78% B; 12–14 min, 78% B; 14–17 min, 78%–95% B; 17–20 min, 95% B; 20–21 min, 95%–5% B; 21–25 min, 5% B. Detection was performed in both positive and negative electrospray ionization (ESI) modes, and the parameter settings were: auxiliary gas (N_2_), 15 Arb; sheath gas (N_2_), 45 Arb; capillary temperature, 330°C; spray voltage, 3.5 kV (ESI^+^), 3.5 kV (ESI^–^); resolution, 120,000 (MS), 60,000 (MS/MS); scan range, *m/z* 100–1,500. The compounds were identified by matching high-accuracy quasi-molecular ion and fragmentation patterns with standard data, and Xcalibur software (version 2.1, Thermo Fisher Scientific, Waltham, MA, United States) was used for data acquisition and analysis.

### Quantitative Analysis of Phenolic Compounds *via* HPLC-DAD-MS

The contents of the identified phenolic compounds in the olive leaves were quantified using a HPLC-DAD-MS system. The polyphenols were separated using a Shimadzu LC-2030C HPLC system (Kyoto, Japan) coupled with a ZORBAX SB-C18 column (4.6 × 250 mm^2^, 5 μm, Agilent Technologies, Savage, MD, United States), and the mobile phases were 1% acetic acid in deionized water (mobile phase A) and acetonitrile (mobile phase B). The following elution conditions were used: 0–11 min, 10%–25% B; 11–16 min, 25%–28.5% B; 16–40 min, 28.5%–90% B; 40–50 min, 90% B; 50–55 min, 90%–10% B; 55–60 min, 10% B, and the flow rate was 1 mL/min. A Thermo Finnigan LCQ DECA mass spectrometer, equipped with an electrospray source, was used for detection, and analyses were performed with scans from 125 to 1,200 *m/z* in negative and positive ion modes. The peaks were identified using an Xcalibur Qual browser, and the quality data and relative retention times of the identified phenolic compounds were compared by UHPLC-Q-Exactive Orbitrap-MS. Then, the spectral peaks were quantitatively analyzed with LabSolutions HPLC software, using calibration curves of the corresponding standard or a compound that contained a similar aglycone (Supplementary Table 1).

### Quantitative Analysis of Triterpenic Acids *via* HPLC-DAD

The triterpenic acid content in the olive leaves was determined using a Shimadzu LC-2030C HPLC system coupled with a ZORBAX SB-C18 column, where the mobile phases consisted of 1% acetic acid in deionized water (A phase) and acetonitrile (B phase). An isocratic elution was implemented using 9% phase A and 91% phase B, and the absorbance was detected at 210 nm. The spectral peaks were quantitatively analyzed with LabSolutions HPLC software, using the calibration curve of the corresponding standard (Supplementary Table 1).

### Determination of Total Flavonoid and Phenol Content

The total flavonoid content (TFC) was determined according to the aluminum chloride colorimetric method (24), where TFC was expressed as milligrams rutin equivalent per gram of dry olive leaf (mg RE/g DW).

The total phenol content (TPC) was determined using the Folin-Ciocalteu method, with spectrophotometric measurements at 765 nm (25). TPC was expressed as milligrams gallic acid equivalent per gram dry olive leaf (mg GAE/g DW).

### Determination of Antioxidant Capacity

The antioxidant activity of the olive leaf extract was determined by DPPH, ABTS, and ferric reducing antioxidant power (FRAP) assays, as previously described in (26). For the DPPH assay, 0.1 mL of sample extract was transferred to a test tube, and 3.9 mL of the 0.1 mM DPPH reaction solution was added. Then, the reaction was allowed to proceed in the dark for 30 min, where the absorbance at 517 nm was measured by a spectrophotometer (UNICO, UV-2600, Shanghai, China). For the ABTS assay, 0.1 mL of sample extract was mixed with 2.9 mL of 7 nM ABTS reaction solution for 5 min at room temperature, and then, the absorbance at 734 nm was measured. The FRAP activities of all of the samples were measured using a FRAP working solution, and the absorbance of the mixture was measured at 593 nm. Subsequently, a Trolox solution was used to establish the standard curve for the antioxidant capacity, which was expressed as milligram Trolox equivalents per gram of dry olive leaf (mg TE/g DW).

### Determination of α-Glucosidase and α-Amylase Inhibition Activities

*In vitro* antidiabetic assays were performed following a previously described method, with acarbose as a standard (27). The α-amylase inhibition reaction mixture consisted of 20 μL of extract, and 20 μL of α-amylase solution (1 units/mL, dissolved in 0.1 M sodium phosphate buffer, pH 6.9), followed by incubation at 37°C for 10 min. Then, the reaction was started by adding 40 μL of starch solution (2 g/L in boiled sodium phosphate buffer). After incubation for 20 min at 37°C, the reaction was stopped by the addition of 80 μL of 0.4 M HCl, followed by 100 μL of iodine reagent solution (5 mM iodine and 5 mM potassium iodide), and the absorbance was recorded at 620 nm.

For the α-glucosidase inhibition assay, 50 μL of the extract was mixed with 50 μL of the α-glucosidase solution (1.5 units/mL, dissolved in 0.05 M phosphate buffer, pH 6.5) and 50 μL of the *p*-nitrophenyl-α-D-glucopyranoside solution (5 mM in phosphate buffer). Then, the mixture was incubated at 37°C for 20 min in the dark. Finally, 100 μL of 0.1 M Na_2_CO_3_ solution was added, and the absorbance was recorded at 405 nm, and the inhibitory activities of α-amylase and α-glucosidase were expressed as equivalents of acarbose (mg ACAEs/g DW) (28).

### Determination of Angiotensin-Converting Enzyme Inhibition Activity

The angiotensin-converting enzyme (ACE) inhibition activity of the olive leaves was determined according to a method described by Wu et al., with slight modification (29). First, 50 μL of the extract was mixed with 125 μL of the substrate (6.5 mM HHL in 50 mM sodium borate buffer containing 0.3 M NaCl, pH 8.3) and incubated at 37°C for 5 min. Afterward, 50 μL of the ACE solution (0.1 units/mL in borate buffer, pH 8.3) was added to the mixture to start the reaction. The mixture was incubated at 37°C for 60 min, then HCl (1 M; 125 μL) was added to the mixture to stop the reaction and 750 μL of ethyl acetate was added for hippuric acid extraction. The samples were then centrifuged at 1,000 rpm for 5 min, and then, 500 μL of the upper layer was collected and evaporated. The hippuric acid residue was dissolved in distilled water, and the absorbance was measured at 228 nm. The inhibitory activity of ACE was expressed as percent inhibition, according to a previously described procedure (29).

### Statistical Analysis

All of the experiments were conducted in triplicate and expressed as the mean ± standard deviation (SD). Statistical analyses were performed with IBM SPSS Software 21 (Chicago, IL, United States) software. Analysis of variance (ANOVA) was performed, where the level of significance was *P* < 0.05.

The contents of 33 phytochemicals were presented by a heat map using the vegan R software package (Version 3.1.2), and principal component analysis was conducted using SIMCA-P (Umetrics, Umea, Sweden). The correlations between the quantification indicators (TPC, TFC, and 33 phytochemicals) and *in vitro* biological activities (DPPH, ABTS, FRAP, α-glucosidase, α-amylase, and ACE inhibition values) were obtained using Spearman’s rank correlations.

## Results and Discussion

### Identification and Quantification of Phytochemicals

A total of 33 phytochemicals were identified in the olive leaves, namely, 19 flavonoids, 2 iridoids, 2 phenylethanoids, 2 coumarins, 2 hydroxycinnamic acids, and 6 triterpenic acids (Table 2 and Supplementary Figure 1). The major classes of phytochemicals (e.g., flavonoids, iridoids, and phenylethanoids) identified in this work agreed with previously reported findings on olive leaves. Martín-García et al. (13) and Lama-Munoz et al. (18) also reported that secoiridoids, flavonoids, and simple phenols were the major class of phytochemicals in the leaves of Picual, Arbequina, and Hojiblanca.

**TABLE 2:** Phytochemical compounds identified in olive leaves *via* UPLC-Q-Exactive Orbitrap-MS.

ID	Rt (min)	Compounds	CAS	Measured m/z	Molecular weight	Molecular formula	MS/MS fragments	Class
1	3.808	Hydroxytyrosol	10597-60-1	153.0550 [M-H]^–^	154.0623	C_8_H_10_O_3_	123.04, 153.05	Phenylethanoids
2	3.809	Hydroxytyrosol 4-*O*-glucoside	54695-80-6	339.1048 [M + Na]^+^	316.1156	C_14_H_20_O_8_	137.06, 179.07, 203.07	Phenylethanoids
3	4.817	Esculin	531-75-9	339.0728 [M-H]^–^	340.0799	C_15_H_16_O_9_	177.02, 339.07	Coumarins
4	5.148	Taxifolin-3-glucoside	27297-45-6	465.1044 [M-H]^–^	466.1118	C_21_H_22_O_12_	125.02, 208.81, 303.05	Flavonoids
5	5.265	Chlorogenic acid	327-97-9	353.0882 [M-H]^–^	354.0955	C_16_H_18_O_9_	191.06, 209.89	Hydroxycinnamic acid
6	5.399	Coumarin	91-64-5	147.0441 [M + H]^+^	146.0368	C_9_H_6_O_2_	91.05, 119.05, 147.04	Coumarins
7	5.698	Secoxyloganin	58822-47-2	403.1250 [M-H]^–^	404.1325	C_17_H_24_O_11_	59.01, 71.01, 89.02	Iridoids
8	5.854	Luteolin-3′,7-di-*O*-glucoside	52187-80-1	609.1474 [M-H]^–^	610.1547	C_27_H_30_O_16_	209.34, 285.04, 447.09	Flavonoids
9	5.982	Plantamajoside	104777-68-6	639.1945 [M-H]^–^	640.2018	C_29_H_36_O_16_	151.04, 161.02, 179.03	Hydroxycinnamic acid
10	6.472	Rutin	153-18-4	609.1475 [M-H]^–^	610.1546	C_27_H_30_O_16_	208.65, 300.03, 301.04	Flavonoids
11	6.698	Quercetin-3-*O*-glucoside	482-35-9	463.0887 [M-H]^–^	464.0961	C_21_H_20_O_12_	300.03, 301.04, 463.09	Flavonoids
12	6.703	Luteolin-7-*O*-glucoside	5373-11-5	449.1077 [M + H]^+^	448.1005	C_21_H_20_O_11_	287.05, 499.11	Flavonoids
13	6.885	Apigenin-7-*O*-neohesperidoside	17306-46-6	577.1571 [M-H]^–^	578.1644	C_27_H_30_O_14_	208.67, 269.05	Flavonoids
14	7.054	Taxifolin	480-18-2	303.0513 [M-H]^–^	304.0586	C_15_H_12_O_7_	125.02, 177.02, 285.04	Flavonoids
15	7.083	Diosmetin-7-*O*-neohesperidoside	38665-01-9	609.1818 [M + H]^+^	608.1745	C_28_H_32_O_15_	301.07, 609.21	Flavonoids
16	7.169	Apigenin-7-*O*-glucoside	578-74-5	431.0988 [M-H]^–^	432.1062	C_21_H_20_O_10_	268.04, 269.05, 431.10	Flavonoids
17	7.187	Kaempferol-7-*O*-glucoside	16290-07-6	447.0941 [M-H]^–^	448.1013	C_21_H_20_O_11_	213.83, 285.04	Flavonoids
18	7.39	Luteolin-4′-*O*-glucoside	6920-38-3	447.0939 [M-H]^–^	448.1013	C_21_H_20_O_11_	210.04, 285.04	Flavonoids
19	7.45	Quercetin-4′-*O*-glucoside	20229-56-5	463.0889 [M-H]^–^	464.0962	C_21_H_20_O_12_	151.00, 178.99, 301.04	Flavonoids
20	7.525	Oleuropein	32619-42-4	563.1733 [M-H]^–^	540.1843	C_25_H_32_O_13_	137.06, 165.05	Iridoids
21	8.427	Tiliroside	20316-62-5	593.1311 [M-H]^–^	594.1384	C_30_H_26_O_13_	145.03, 213.79, 285.04	Flavonoids
22	8.835	Eriodictyol	552-58-9	289.0705 [M + H]^+^	288.0632	C_15_H_12_O_6_	152.02, 163.04, 285.07	Flavonoids
23	8.887	Luteolin	491-70-3	285.0408 [M-H]^–^	286.0481	C_15_H_10_O_6_	59.01, 163.64, 285.04	Flavonoids
24	8.89	Quercetin	117-39-5	301.0356 [M-H]^–^	302.043	C_15_H_10_O_7_	59.01, 151.00, 301.04	Flavonoids
25	8.98	Kaempferol	520-18-3	287.0549 [M + H]^+^	286.0476	C_15_H_10_O_6_	153.02, 287.05	Flavonoids
26	9.699	Apigenin	520-36-5	271.0599 [M + H]^+^	270.0527	C_15_H_10_O_5_	169.64, 271.06	Flavonoids
27	9.84	Hispidulin	1447-88-7	299.0564 [M-H]^–^	300.0637	C_16_H_12_O_6_	284.03, 299.06	Flavonoids
28	11.82	Asiatic acid	464-92-6	487.3435 [M-H]^–^	488.3509	C_30_H_48_O_5_	134.15, 487.34	Triterpenic acids
29	13.02	Oleanonic acid	17990-42-0	455.3517 [M + H]^+^	454.3445	C_30_H_46_O_3_	111.08, 203.18, 455.35	Triterpenic acids
30	13.382	Maslinic acid	4373-41-5	473.3622 [M + H]^+^	472.3551	C_30_H_48_O_4_	203.18, 215.18, 409.35	Triterpenic acids
31	13.505	Corosolic acid	4547-24-4	473.3625 [M + H]^+^	472.3551	C_30_H_48_O_4_	189.16, 205.16, 409.35	Triterpenic acids
32	16.445	Oleanolic acid	508-02-1	455.3537 [M-H]^–^	456.3611	C_30_H_48_O_3_	214.73, 455.35	Triterpenoids
33	16.498	Ursolic acid	77-52-1	457.3673 [M + H]^+^	456.3601	C_30_H_48_O_3_	163.15, 411.36, 439.36	Triterpenoids

*Rt, retention time.*

The concentrations of individual phytochemicals are presented in Table 3, which were obtained through calculations based on the curves of their corresponding standards, or standards with a similar aglycone. Iridoids were the dominant phytochemical groups identified in the olive leaves, and their concentrations ranged from 566 (Canino, fresh) to 22,096 mg/kg DW (Nevadillo fino, HD), accounting for 7.8–49.63% of all of the identified phytochemicals (Table 3). Oleuropein was the most abundant iridoid in the olive leaves, with content values of 524–21,189 mg/kg DW. These results were consistent with a report by Benavente-Garcia et al. (8), which showed that oleuropein was the dominant compound in olive leaves, accounting for 24.54% of the total phenolics. Flavonoids were the second-most abundant compound, and their concentration varied between 2,027 (Canino, fresh) and 8,055 mg/kg DW (Nevadillo fino, FD), accounting for 11.62–17.82% of the total phytochemicals. Some flavonoid aglycones identified in previous studies were also detected in our study, such as luteolin, quercetin, kaempferol, and apigenin (13, 30). Three flavonoids, namely, taxifolin, eriodictyol, and hispidulin, were reported for the first time. However, most of the identified flavonoids were flavonoid glycosides, including derivatives of diosmetin, taxifolin, quercetin, kaempferol luteolin, and apigenin. Among the flavonoid glycosides, luteolin and its glycosides derivatives (i.e., luteolin-7-*O*-glucoside, luteolin-3′,7-di-*O*-glucoside, and luteolin-4′-*O*-glucoside) were dominant, followed by kaempferol, apigenin, quercetin, and their corresponding glycosides derivatives (i.e., kaempferol-7-*O*-glucoside, apigenin-7-*O*-glucoside, quercetin-3-*O*-glucoside, and quercetin-4′-*O*-glucoside). As previously mentioned, flavonoids were the most diverse class of phenolics in olive leaves (2, 13, 31). Our results demonstrated that olive leaves were a rich source of flavonoids, especially flavonoid *O*-glycosides. In addition to iridoids and flavonoids, two coumarins, hydroxycinnamic acids, and phenylethanoids were identified. As previously reported, hydroxytyrosol was the main component of simple phenols and an important compound in the formation of oleuropein (1).

**TABLE 3:** Concentrations of individual phytochemicals identified in olive leaves.

Compounds	Nevadillo fino			Canino			Huaou5			I79		
	Fr	HD	FD	Fr	HD	FD	Fr	HD	FD	Fr	HD	FD
Hydroxytyrosol	138.59 ± 6.85h	303.84 ± 1.96f	496.45 ± 12.20d	66.82 ± 0.76i	267.78 ± 8.00g	879.33 ± 34.94b	72.41 ± 2.70i	370.34 ± 2.84e	1,489.16 ± 1.41a	114.30 ± 3.68h	367.41 ± 0.60e	756.65 ± 4.03c
Hydroxytyrosol 4-*O*-glucoside	ND	ND	ND	ND	54.23 ± 10.28b	110.16 ± 6.85a	ND	ND	ND	ND	ND	ND
*Total phenylethanoids*	138.59 ± 6.85g	303.84 ± 1.96f	496.45 ± 12.20d	66.82 ± 0.76h	322.01 ± 18.28f	989.49 ± 28.09b	72.41 ± 2.70h	370.34 ± 2.84e	1,489.16 ± 1.41a	114.3 ± 3.68g	367.41 ± 0.60e	756.65 ± 4.03c
Esculin	1.83 ± 0.04g	13.65 ± 0.11c	15.32 ± 0.38b	0.70 ± 0.03h	15.16 ± 0.57b	5.47 ± 0.02e	1.33 ± 0.17gh	4.04 ± 0.09f	20.42 ± 0.26a	1.01 ± 0.05h	20.91 ± 0.13a	7.49 ± 0.20d
Coumarin	5.04 ± 0.39g	16.08 ± 0.90b	14.91 ± 0.29bc	8.19 ± 0.65f	13.16 ± 0.68de	11.07 ± 1.07e	4.89 ± 0.79g	19.92 ± 0.23a	12.99 ± 0.94d	4.02 ± 0.08g	14.97 ± 0.51bc	8.42 ± 0.15f
*Total coumarins*	6.88 ± 0.43fg	29.73 ± 1.01c	30.23 ± 0.67c	8.88 ± 0.68f	28.31 ± 1.26c	16.54 ± 1.09e	6.22 ± 0.96g	23.95 ± 0.15d	33.40 ± 1.2b	5.03 ± 0.03g	35.88 ± 0.39a	15.9 ± 0.35e
Chlorogenic acid	0.52 ± 0.35f	2.45 ± 0.68dce	3.21 ± 0.33cd	1.37 ± 0.36ef	7.70 ± 0.22a	8.97 ± 1.29a	1.81 ± 0.21def	0.63 ± 0.42f	2.60 ± 0.46cd	2.18 ± 0.19de	3.72 ± 0.10c	5.39 ± 0.46b
Plantamajoside	ND	ND	ND	ND	ND	ND	ND	ND	ND	13.46 ± 6.06	ND	ND
*Total hydroxycinnamic acid*	0.52 ± 0.35d	2.45 ± 0.68cd	3.21 ± 0.33cd	1.37 ± 0.36d	7.70 ± 0.22bc	8.97 ± 1.29b	1.81 ± 0.21d	0.63 ± 0.42d	2.60 ± 0.46cd	15.63 ± 6.24a	3.72 ± 0.10bcd	5.39 ± 0.46bcd
Taxifolin-3-glucoside	4.36 ± 0.78f	15.10 ± 0.57c	21.97 ± 2.90b	12.53 ± 1.52cd	24.36 ± 0.70ab	28.90 ± 3.97a	7.50 ± 0.69ef	14.81 ± 0.84c	19.89 ± 0.28b	8.06 ± 0.06def	12.14 ± 0.84cd	11.94 ± 0.17cde
Luteolin-3′,7-di-*O*-glucoside	64.45 ± 4.61d	280.95 ± 14.75a	313.82 ± 10.58a	67.09 ± 1.55d	148.85 ± 2.45c	168.91 ± 3.79c	63.69 ± 2.44d	224.16 ± 17.94b	186.40 ± 48.00bc	70.56 ± 5.11d	154.22 ± 7.82c	157.14 ± 8.36c
Rutin	380.31 ± 4.45c	558.69 ± 17.40b	853.17 ± 10.79a	72.14 ± 3.55i	340.56 ± 6.99d	358.59 ± 10.46cd	120.68 ± 2.36h	237.07 ± 8.57e	537.69 ± 0.35b	52.45 ± 0.90i	152.76 ± 0.49g	184.15 ± 0.12f
Quercetin-3-*O*-glucoside	64.79 ± 13.82g	155.25 ± 26.64d	156.19 ± 2.14d	82.85 ± 14.09fg	102.36 ± 9.33ef	122.60 ± 5.34e	93.32 ± 11.91ef	166.79 ± 2.42cd	197.88 ± 3.91bc	120.39 ± 1.30e	218.03 ± 2.86b	316.49 ± 7.57a
Luteolin-7-*O*-glucoside	876.85 ± 20.11c	2,831.55 ± 521.78ab	2,539.70 ± 30.35ab	585.10 ± 23.22c	2,089.00 ± 45.74ab	1,987.59 ± 194.58b	570.93 ± 32.87c	2,289.58 ± 17.43ab	1,988.72 ± 599.61b	856.80 ± 31.49c	2,882.52 ± 4.76a	2,728.48 ± 560.42ab
Apigenin-7-*O*-neohesperidoside	249.01 ± 1.83e	331.60 ± 8.87d	345.77 ± 2.90cd	269.13 ± 10.32e	250.00 ± 8.98e	263.52 ± 15.75e	503.97 ± 5.77a	362.32 ± 6.09c	396.47 ± 0.38b	227.75 ± 2.23f	182.71 ± 3.18g	189.30 ± 0.57g
Taxifolin	143.26 ± 2.97e	27.93 ± 0.35i	78.55 ± 3.61f	43.55 ± 2.00h	159.05 ± 4.13d	194.03 ± 6.24c	55.67 ± 0.24g	50.61 ± 0.13gh	210.24 ± 0.10b	144.14 ± 6.61e	42.46 ± 0.26h	274.30 ± 3.42a
Diosmetin-7-*O*-neohesperidoside	2.51 ± 0.09h	8.99 ± 0.20b	12.67 ± 0.29a	3.34 ± 0.15g	3.66 ± 0.21fg	4.55 ± 0.38e	2.11 ± 0.02h	5.94 ± 0.30d	6.82 ± 0.12c	2.24 ± 0.10h	3.54 ± 0.06fg	4.02 ± 0.06ef
Apigenin-7-*O*-glucoside	242.66 ± 5.77c	335.35 ± 4.53a	284.35 ± 1.58b	78.77 ± 3.03f	145.01 ± 5.26e	138.96 ± 2.36e	235.19 ± 8.44c	321.76 ± 6.96a	194.54 ± 1.07d	136.00 ± 1.80e	241.98 ± 7.49c	183.50 ± 1.90d
Kaempferol-7-*O*-glucoside	469.51 ± 8.35f	2,128.62 ± 14.78c	2,530.56 ± 261.22b	621.88 ± 14.36ef	2,378.35 ± 74.87b	2,462.51 ± 21.22b	614.87 ± 11.89ef	2,072.19 ± 32.7c	1,638.05 ± 29.50d	744.85 ± 17.55f	2,681.13 ± 11.21a	2,665.46 ± 7.19a
Luteolin-4′-*O*-glucoside	39.70 ± 3.83g	155.31 ± 2.85cd	171.14 ± 4.03c	57.38 ± 5.85gf	233.01 ± 19.88b	271.62 ± 8.02a	41.59 ± 3.29g	78.19 ± 1.38f	120.36 ± 5.73e	54.58 ± 3.39g	133.39 ± 0.55de	122.08 ± 11.76e
Quercetin-4′-*O*-glucoside	4.89 ± 1.10b	18.31 ± 1.62a	18.25 ± 0.38a	ND	ND	ND	ND	ND	5.06 ± 0.81b	3.20 ± 0.47bc	ND	1.81 ± 0.09cd
Tiliroside	15.34 ± 0.15b	18.54 ± 0.18a	13.55 ± 0.22c	6.61 ± 0.18g	4.76 ± 0.13h	7.39 ± 0.45f	6.67 ± 0.14g	9.17 ± 0.16e	9.18 ± 0.01e	6.75 ± 0.05g	9.93 ± 0.44d	15.35 ± 0.43b
Eriodictyol	141.39 ± 4.43d	286.98 ± 4.28b	405.71 ± 57.8a	58.38 ± 1.52ef	190.46 ± 8.08cd	249.21 ± 57.83bc	41.28 ± 1.17f	132.09 ± 1.59de	447.72 ± 34.66a	61.28 ± 0.72ef	194.35 ± 2.38cd	302.00 ± 7.95b
Luteolin	196.68 ± 9.77d	26.38 ± 0.69i	261.69 ± 6.01c	61.85 ± 2.70fg	29.55 ± 0.19i	185.64 ± 3.46d	70.43 ± 0.37f	49.04 ± 0.50h	488.78 ± 0.66b	87.51 ± 2.39e	55.14 ± 0.84gh	553.45 ± 7.18a
Quercetin	9.21 ± 0.34e	17.05 ± 0.32ab	18.44 ± 1.81a	ND	11.91 ± 0.29d	18.14 ± 0.52a	7.26 ± 0.09f	15.52 ± 0.59bc	18.86 ± 0.48a	ND	13.84 ± 0.16c	18.55 ± 0.54a
Kaempferol	11.83 ± 0.10a	ND	4.91 ± 0.04c	2.39 ± 0.02e	ND	2.64 ± 0.03d	2.19 ± 0.02f	ND	6.02 ± 0.10b	ND	ND	ND
Apigenin	8.83 ± 0.27f	1.61 ± 0.07i	20.96 ± 0.53c	4.19 ± 0.20h	1.73 ± 0.04i	5.37 ± 0.01g	11.69 ± 0.04e	ND	36.07 ± 0.11a	5.45 ± 0.16g	13.04 ± 0.12d	23.32 ± 1.04b
Hispidulin	1.32 ± 0.05cd	0.36 ± 0.13e	4.24 ± 0.60a	0.72 ± 0.04de	0.17 ± 0.02e	1.410 ± 0.02	1.15 ± 0.08	0.42 ± 0.20e	3.18 ± 0.40b	1.33 ± 0.05cd	0.30 ± 0.07e	4.42 ± 0.14a
*Total flavonoids*	2,926.91 ± 27.78e	7,198.57 ± 574.75abc	8,055.62 ± 220.02a	2,027.90 ± 34.20e	6,112.77 ± 181.84d	6,471.56 ± 221.9cd	2,450.17 ± 48.34e	6,029.63 ± 93.85d	6,511.89 ± 713.25cd	2,583.35 ± 57.87e	6,991.45 ± 4.48bcd	7,755.76 ± 553.73ab
Secoxyloganin	70.21 ± 1.05gi	906.28 ± 3.14b	203.59 ± 4.63e	41.16 ± 1.57hi	1,383.22 ± 34.26a	346.67 ± 7.24d	21.64 ± 0.78i	759.54 ± 3.11c	132.46 ± 0.50f	18.03 ± 0.11i	737.54 ± 9.91c	87.15 ± 3.20g
Oleuropein	1,134.89 ± 34.95i	21,189.85 ± 2.16a	5,681.83 ± 135.30f	524.89 ± 23.13j	16,380.58 ± 480.39c	7,318.12 ± 401.71e	646.41 ± 27.86ij	13,248.99 ± 168.76d	3,126.35 ± 83.26h	590.14 ± 23.51ij	17,699.43 ± 158.23b	4,004.78 ± 124.39g
*Total iridoids*	1,205.10 ± 33.9i	22,096.13 ± 0.98a	5,885.42 ± 139.93f	566.05 ± 24.70i	17,763.8 ± 514.66c	7,664.79 ± 408.96e	668.05 ± 28.64ij	14,008.53 ± 171.87d	3,258.81 ± 83.76h	608.17 ± 23.62ij	18,436.97 ± 168.14b	4,091.93 ± 127.59g
Asiatic acid	22.99 ± 0.32b	23.92 ± 4.91ab	23.74 ± 1.43ab	17.66 ± 0.43cd	9.06 ± 0.25g	8.78 ± 1.19g	15.28 ± 0.20f	27.96 ± 1.86a	28.77 ± 1.45a	18.55 ± 1.19bcd	15.92 ± 0.55ef	16.91 ± 1.03de
Oleanonic acid	1,669.26 ± 25.47c	723.32 ± 69.44ef	827.32 ± 90.63d	721.7 ± 42.74ef	774.95 ± 85.53e	838.83 ± 44.77d	681.45 ± 54.52g	2,252.43 ± 102.52b	2,267.04 ± 42.56a	709.13 ± 32.25f	848.88 ± 20.11d	907.88 ± 88.96d
Maslinic acid	4,315.21 ± 104.48b	3,712.34 ± 254.18d	4,420.64 ± 378.32b	3,235.5 ± 263.79e	5,929.92 ± 91.33a	6,071.36 ± 727.56a	4,109.96 ± 392.19cd	4,384.59 ± 304.27b	4,369.35 ± 83.53b	4,124.62 ± 138.93b	4,489.45 ± 112.45b	4,455.58 ± 43.24b
Corosolic acid	1,527.29 ± 79.99d	1,974.92 ± 145.19b	2,274 ± 73.65a	1,620.68 ± 118.9cd	928.04 ± 69.12f	519.88 ± 60.15g	1,348.02 ± 13.88e	1,450.35 ± 33.96de	1,707.25 ± 51.15c	1,518.71 ± 11.15cd	1,591.59 ± 63.74cd	1,538.47 ± 55.33cd
Oleanolic acid	8,428.8 ± 622.05c	8,231.99 ± 495.41bc	10,227.14 ± 442.99a	8,012.36 ± 793.33bc	9,687.59 ± 516.28a	10,455.69 ± 313.77a	7,584.04 ± 414.18c	8,552.62 ± 242.16b	10,476.67 ± 125.49a	8,090.84 ± 197.36c	8,439.63 ± 122.94c	10,111.41 ± 252.52a
Ursolic acid	201.42 ± 17.33bc	206.01 ± 25.32bc	258.86 ± 11.83a	216.55 ± 31.80b	146.35 ± 14.08e	189.39 ± 8.43cd	185.85 ± 14.68d	213.19 ± 30.55b	207.05 ± 16.76bc	190.42 ± 8.52cd	192.65 ± 16.72d	204.47 ± 12.68bc
*Total triterpenic acids*	16,164.97 ± 923.9b	14,872.5 ± 1,045.4c	18,031.7 ± 1,041.32a	13,824.45 ± 1,341.7c	17,475.91 ± 887.30b	18,083.93 ± 1,245.56a	13,924.6 ± 930.67c	16,881.14 ± 801.6b	19,056.13 ± 384.35a	14,652.27 ± 474.2b	15,578.12 ± 453.48b	17,234.72 ± 631.74a

*Fr, HD, and FD denote fresh, hot air-dried, and freeze-dried olive leaf samples, respectively. Data are expressed as mean ± standard deviation (n = 3) in mg/kg dry weight (DW). For each line, mean values followed by different letters indicate a significant difference (P < 0.05). ND denotes not detected.*

In addition to phenolic compounds, triterpene compounds (i.e., Asiatic, oleanonic, maslinic, corosolic, ursolic, and oleanolic acids) also accounted for a large proportion of phytochemicals found in the olive leaves (Table 3). Olive products have been reported to contain high amounts of triterpenic acids. Moreno-González et al. (32) reported that table olives of Arbequina and Empeltre varieties were especially rich in maslinic acids (1.86–2.51 g/kg) and oleanolic acids (0.78–0.90 g/kg), and small amounts of ursolic acid (13.2 mg/kg) were found by Kalogeropoulos et al. (33) in virgin olive oil. However, few studies have reported the occurrence of triterpenic acids in olive leaves. In this study, triterpenic acids were identified by the UPLC-Q-Exactive Orbitrap-MS method. In addition, we found that triterpenic acids were abundant in olive leaves, with the total content ranging between 13,824 (Canino, fresh) and 19,056 mg/kg DW (Huaou5, FD).

Bioactive compounds in plants will be affected not only by cultivar/genotype, developmental processes, and environmental factors during plant growth (17, 34) but also subsequent processing conditions, such as the drying conditions (35). This study investigated the effects of two drying processes on the quantities of individual phytochemical compounds present in olive leaves from four cultivars. The total concentrations of phytochemicals in the fresh samples (16,495.47–20,442.97 mg/kg DW) were significantly lower than the freeze-dried samples (29,860.35–36,235.28 mg/kg DW) and hot air-dried samples (37,314.28–44,503.22 mg/kg DW, Table 3). For all of the assayed cultivars, the drying process caused a significant increase (*P* < 0.05) in the phenolic content of the olive leaf extracts. These results were inconsistent with the fact that the phenolic compounds were modified or degraded during the drying processes (36). This was attributed to the different phenolic profiles of the raw materials used and the different stress sensitivities of each polyphenol to the drying conditions (37). Polat et al. (36) revealed that HD and FD treatments caused approximately 70% loss of anthocyanins in black carrot pomace, which was explained by the fact that the anthocyanins easily degraded into smaller molecules such as aldehydes and benzoic acid during dehydration. However, the stability of the polyphenols identified in olive leaves, such as flavonoid glycosides and oleuropein, was relatively high (38). In addition, different drying conditions, such as temperature, may have caused these inconsistent results. Vidinamo et al. (39) concluded that the contents of phytochemicals were usually increased after thermal drying at high temperatures above 60°C. During the drying processes, the activation of oxidative enzymes, such as polyphenol oxidase and peroxidase, possibly led to a loss of phenolic compounds (39). However, thermal processing at high temperatures has also been shown to inactivate these enzymes (40). In this study, the olive leaves were oven-dried at 105°C, which likely deactivated enzymatic oxidation, thus avoiding phenolic degradation. Additionally, HD and FD could break down the cellular constituents through high temperature stress or ice crystals, which released the bound phenolics from the plant cell walls (41). Taking the above into consideration, we supposed that the increase in extractable phenolics in the dried olive leaves could be attributed to the phenolic stability, release of bound phenolics, and inactivation of oxidase and peroxidase in the olive leaves.

**FIGURE 1:**
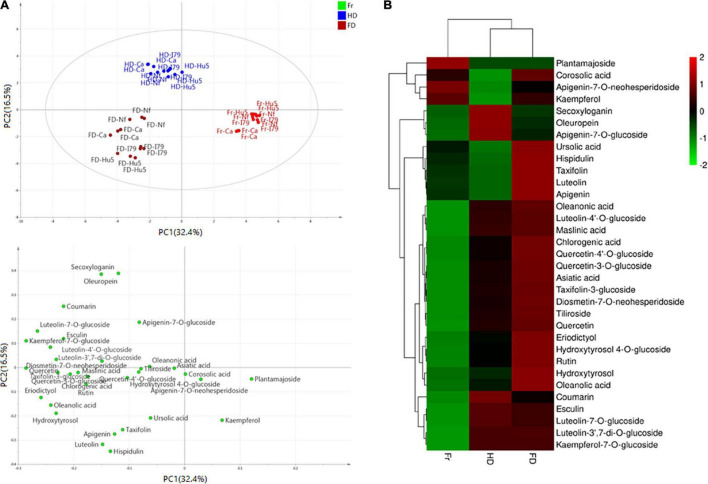
**(A)** Principal component analysis results of olive leaf samples according to their phytochemical profiles (score plots and loading plot). **(B)** Clustered heat map analysis of the 33 individual phytochemical compounds in the fresh and dried olive leaf samples. Red color indicates major abundance, green color indicates minor abundance, and Fr, HD, and FD denote fresh, hot air-dried, and freeze-dried olive leaf samples, respectively.

Furthermore, the principal component analysis and clustered heat map analysis revealed the phytochemical profiles of the dried olive leaves of each cultivar. As shown in Figure 1A, 33 compounds were analyzed, and the first four principal components (PCs) explained 73.4% of the total variance (PC1 32.4%, PC2 16.5%, PC3 14.4%, and PC4 10.1%). Olive leaf samples were divided into fresh, FD, and HD groups, based on their drying methods. For the four cultivars, fresh olive leaves were separated from most of the dried samples by PC1, and they were identified as the poorest source of polyphenols. The HD and FD groups, at the opposite sides of the PC2 coordinates, showed different phytochemical patterns. These results were further confirmed through clustered heat map analysis (Figure 1B). On the horizontal axis of the heat map, FD and HD were clustered into a group, while fresh olive leaves belonged to the other cluster. The HD group exhibited a higher level of iridoids (i.e., secoxyloganin and oleuropein), and the FD group exhibited higher content of flavonoid aglycones (i.e., luteolin, quercetin, kaempferol, apigenin, hispidulin, eriodictyol, and taxifolin) and hydroxytyrosol (Figure 1B). The different phytochemical profiles were attributed to the thermal sensitivities of the different compounds, as oleuropein was shown to exhibit good thermal stability at temperatures up to 130°C (38). Similar findings have been reported on Spanish olive leaves, and Hussam et al. (20) found that air drying at high temperatures improved the oleuropein content of the extracts. By contrast, non-glycoside polyphenols (e.g., quercetin, luteolin, and chlorogenic acid) easily degraded under thermal processing (42). Consistent with previous work, the flavonoid aglycone concentration of the HD extracts was half that of the fresh extracts. Interestingly, there were no significant (Table 3, *P* > 0.05) differences between most of the flavonoid glycosides (e.g., kaempferol-7-*O*-glucoside, apigenin-7-*O*-neohesperidoside, and luteolin-3′,7-di-*O*-glucoside) in the HD and FD samples for cultivars Canino and I79. As previously reported, polyphenols existing as glycosides were shown to be more resistant to pH, heat, and other ecological factors than the non-glycosides, because the prevention of nucleophilic attack made the former more resistant to degradation (23).

### Total Flavonoid Content and Total Phenol Content

**FIGURE 2:**
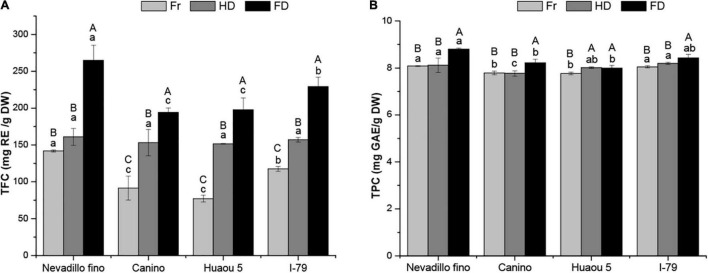
TFC **(A)** and TPC **(B)** of dried olive leaves from four cultivars. Fr, HD, and FD denote fresh, hot air-dried, and freeze-dried olive leaf samples, respectively. Data are expressed as mean ± standard deviation (*n* = 3). Different letters in the different treatments (A to C) and cultivars (a to c) indicate a significant difference (*P* < 0.05).

Typically, phenolic compounds identified *via* mass spectrometry will not represent all of the polyphenols. Accordingly, we investigated the TFC and TPC in the olive leaves using aluminum chloride colorimetric and Folin-Ciocalteu assay techniques, respectively. The TFC and TPC in the olive leaves were 77.25–264.62 and 7.76–8.79 mg GAE/g DW, respectively (Figure 2). The data obtained in this study were within the ranges reported in the literature. Similar TPC and TFC ranges have been reported for Frantoio olive leaves (TPC: 21.6–106.9 mg GAE/g DW, and TFC: 49.4–871.5 mg RE/g DW) (12). Moreover, the TFC content in the olive leaves was considerably higher than other agro-industrial byproducts, including tangerine pomace (40.70 mg RT/g), grape canes (31.9 mg RT/g), and mango byproducts (4.76 mg RT/g) (43).

The drying process enhanced the release of extractable phenolics. As shown in Figure 2, the FD and HD samples exhibited significantly higher TFC and TPC (*P* < 0.05) content than the fresh olive leaves (77.26–141.97 mg RE/g DW and 7.76–8.08 mg GAE/g DW, respectively), and these results were consistent with the variations in total phytochemical content. Comparing the different drying processes, the FD samples exhibited significantly higher TFC and TPC content than the HD samples for all of the studied cultivars (except for the TPC of Huaou5). For example, the TFC values of the FD and HD Nevadillo fino samples were 264.62 and 161.04 mg RE/g DW, respectively. Our results consistently indicated that hot air-drying led to a loss of phenolics, especially flavonoid aglycones. However, a previous study reported that Frantoio olive leaves oven dried at 105°C exhibited higher TFC content (871.5 vs. 679.6 mg RE/g DW) and TPC (106.9 vs. 91.2 mg GAE/g DW) than the freeze-dried samples (12). This contradictory conclusion was possibly explained by the differences in olive variety, drying time, and extraction method (solvent type, solvent concentration, and extraction temperature). In a previous study, the olive leaves were subjected to longer drying times (180 vs. 90 min), which possibly led to the release of more bound phenolics from the breakdown of cellular constituents (40). Furthermore, as reported by Lachowicz et al. (44), the initial chemical compositions of different saskatoon berry cultivars potentially influenced the alterations of the polyphenolic compounds during convective drying. Therefore, these inconsistent results might also be correlated with the used cultivars, as the phytochemical compounds in the olive leaves differed among the different cultivars (45). Moreover, ultrasound-assisted extraction was employed to recover the phenolic compounds in this study, which potentially influenced phenolic extraction (30).

### Bioactive Potential of Olive Leaves

#### Antioxidant Activity

The antioxidant activities of the olive leaf extracts from the two drying processes were evaluated by three complementary methods (DPPH, ABTS, and FRAP assays). The antioxidant activities of the olive leaves differed among the cultivars (Table 4). Fresh Nevadillo fino showed the highest DPPH radical scavenging ability (423.65 mg TE/g DW), while fresh I79 had the highest ABTS and FRAP assay values (483.21 and 531.35 mg TE/g DW, respectively). Fresh Huaou5 showed the lowest DPPH, ABTS, and FRAP values (324.52, 444.12, and 428.52 mg TE/g DW, respectively). These results were within the reported ranges for olive leaves, as the ABTS and DPPH antiradical activities of the olive leaves ranged from 61.05 to 335.5 mg TE/g DW and 42.7–378.2 mg TE/g DW, respectively (2, 12, 18). The results of this study, as well as other studies, confirmed that the olive leaf extracts had excellent antioxidant properties compared to other agro-food residues. For example, much lower antioxidant activity was found in Portuguese vine shoot waste (35.3 mg TE/g DW for DPPH) (46), raspberry pomace (27.45 mg TE/g DW for DPPH) (47), and wheat bran (43.43 and 129.50 TE/g extract for DPPH and ABTS, respectively) (48). Moreover, the FD samples of all of the studied cultivars exhibited the highest FRAP, DPPH, and ABTS radical scavenging activities (Table 4). The antioxidant activity was synergistically promoted by the phenolics present in the samples and the reduction capacity of the matrix (30). Generally, a direct relationship has been found between the phenolic content and antioxidant activity (42). The higher antioxidant activities of the FD and HD samples were attributed to their higher TPC and TFC content, compared to the fresh samples.

**TABLE 4:** Antioxidant, α-amylase, α-glucosidase, and ACE inhibition activities of the olive leaves.

Samples	DPPH mg TE/g DW	FRAP mg TE/g DW	ABTS mg TE/g DW	α -amylase inhibition mg ACAE/g DW	α -Glucosidase inhibition mg ACAE/g DW	ACE inhibition %
Nevadillo fino-Fr	423.65 ± 22.74^cd^	486.67 ± 6.21^cd^	428.01 ± 5.32^d^	82.12 ± 1.94^bcde^	537.57 ± 70.36^f^	17.56 ± 2.36^e^
Nevadillo fino-HD	433.65 ± 9.24^cd^	490.39 ± 18.44^cd^	427.53 ± 7.23^d^	59.75 ± 4.66^de^	479.79 ± 95.44^f^	42.06 ± 4.05^cd^
Nevadillo fino-FD	683.43 ± 33.86^a^	604.87 ± 32.46^ab^	603.13 ± 7.45^a^	231.8 ± 5.07^a^	6,352.99 ± 109.22^a^	81.99 ± 14.81^a^
Canino-Fr	399.27 ± 45.39^d^	485.13 ± 30.14^cd^	435.22 ± 20.89^cd^	107.27 ± 48.47^bcd^	1,253.45 ± 61.14^de^	56.76 ± 3.19^b^
Canino-HD	443.44 ± 54.01^cd^	529.29 ± 96.10^c^	478.64 ± 34.46^c^	39.76 ± 4.69^e^	561.68 ± 88.51^f^	48.08 ± 1.32^bc^
Canino-FD	688.06 ± 45.48^a^	608.84 ± 6.76^ab^	524.54 ± 33.24^b^	133.93 ± 51.73^b^	3,538.80 ± 192.98^b^	81.31 ± 1.04^a^
Huaou5-Fr	324.52 ± 19.31^e^	444.12 ± 1.78^d^	428.52 ± 19.21^d^	69.05 ± 30.01^cde^	491.00 ± 19.41^f^	17.12 ± 6.57^e^
Huaou5-HD	463.57 ± 19.38^c^	567.07 ± 13.09^bc^	483.33 ± 6.76^bc^	37.73 ± 2.27^e^	456.04 ± 14.24^f^	34.02 ± 1.79^d^
Huaou5-FD	577.56 ± 60.34^b^	616.61 ± 27.38^ab^	561.21 ± 40.61^ab^	183.04 ± 29.30^ab^	1,566.82 ± 286.39^cd^	77.75 ± 10.16^a^
I79-Fr	414.50 ± 19.95^cd^	531.35 ± 52.13^c^	483.21 ± 18.04^bc^	118.58 ± 26.11^bc^	675.12 ± 1.44^ef^	54.98 ± 1.92^b^
I79-HD	543.94 ± 22.30^b^	589.42 ± 28.73^abc^	491.30 ± 33.12^bc^	59.37 ± 12.02^de^	358.63 ± 30.75^f^	50.87 ± 7.58^bc^
I79-FD	742.93 ± 53.44^a^	634.85 ± 26.30^a^	591.16 ± 9.94^a^	231.12 ± 28.91^a^	1,916.63 ± 252.71^c^	87.45 ± 11.87^a^

*Fr, HD, and FD denote fresh, hot air-dried, and freeze-dried olive leaf samples, respectively. TE, Trolox equivalent; ACAE, acarbose equivalent. Data are expressed as mean ± standard deviation (n = 3) in mg/kg dry weight (DW). For each line, mean values followed by different letters indicate significant difference (P < 0.05).*

#### α-Amylase and α-Glucosidase Inhibitory Activities

Polyphenol-rich diets can suppress the production and absorption of glucose from the gastrointestinal tract, as they can bind non-covalently into the active site residues of α-amylase and α-glucosidase (15). Therefore, plant-based inhibitors of α-amylase and α-glucosidase have received increasing attention for controlling diabetic problems (49), and a study on the α-amylase and α-glucosidase inhibition activities of olive leaf phenolic extracts was vital for evaluating the potential glycemic control of the extracts. The results of α-amylase and α-glucosidase inhibition are presented in Table 4. The olive leaf extracts showed considerable inhibition potential for the two enzymes. Among all of the tested cultivars, the FD samples were the most effective against α-amylase (133.93–231.80 mg ACAE/g), followed by the fresh samples (69.05–118.58 mg ACAE/g). Similarly, the FD samples showed the highest α-glucosidase enzyme inhibition activity, which was 2.82–13.26 times greater than the fresh samples (Table 4). The HD samples showed the least activity toward α-amylase (37.73–59.37 mg ACAE/g) and α-glucosidase (358.63–561.68 mg ACAE/g), despite their higher TFC and TPC content compared to the fresh samples. The starch digestive enzymes inhibition potential of the olive leaves was likely related to the presence of specific flavonoids (27). In addition, the differences in α-amylase and α-glucosidase inhibition were explained by the selectively inhibitory effects of the flavonoids (50). Previous studies have shown that the inhibitory effects of flavonoids were mainly dependent on their specific chemical structures, and flavonoids with double bonds between the C2 and C3 of the C-ring appeared to be particularly important for the inhibition of α-amylase, whereas the hydroxyl group at the C3 of the flavonoid C-ring was related to inhibition of α-glucosidases (15).

#### Angiotensin-Converting Enzyme-Inhibitory Activity

Angiotensin-converting enzyme inhibition is important for downregulating blood pressure because ACE stimulates the conversion of angiotensin I to angiotensin II, a strong vasoconstrictor that can upregulate blood pressure and cause hypertension (51). Olive leaves have been commonly used as a folk medicine for antihypertension (1); however, the role of ACE inhibition in the antihypertension effect of olive leaf extracts has not been investigated. Most of the olive leaf extracts, especially the FD samples, exhibited efficient ACE inhibition (Table 4). For the four olive-leaf cultivars, the FD samples showed the highest ACE inhibition rate, reaching 77.75–87.45%. The ACE inhibition rates of the fresh samples from Nevadillo fino and Huaou5 cultivars were the lowest (17.12–17.56%). For the Nevadillo fino and Huaou5 samples, ACE inhibition followed the order of FD > HD > fresh leaves, whereas for the Canino and I79 cultivars, the order was FD > fresh leaves > HD. This discrepancy in ACE inhibition activity was likely related to the difference in the phenolic composition of the extracts, which varied by cultivar and drying process.

### Correlation Between Phytochemicals and Bioactive Potentials

**FIGURE 3:**
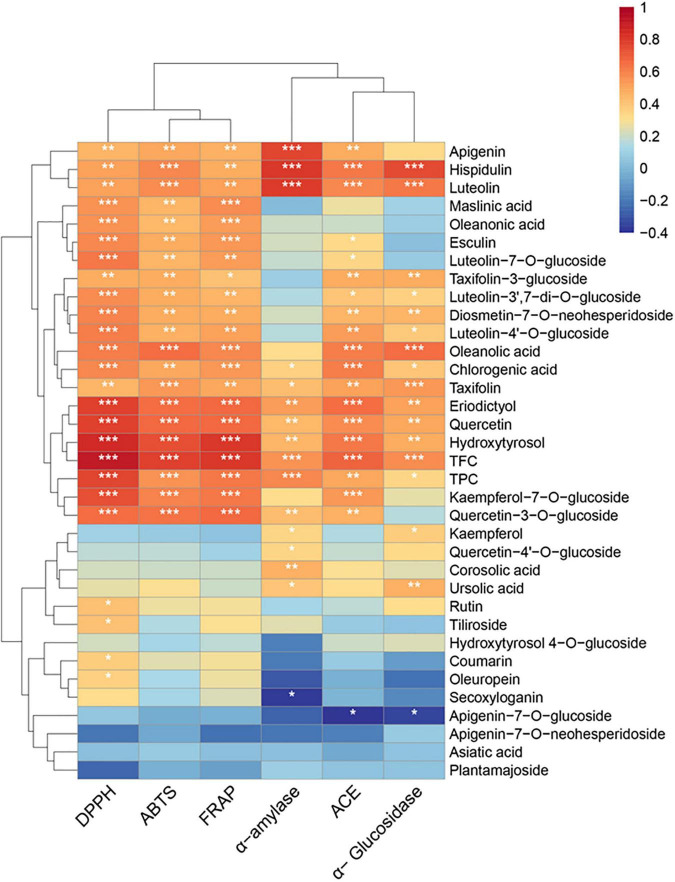
Heat map of Spearman’s correlation between chemical constituents and the bioactivities (DPPH; ABTS; FRAP; α-amylase, α-glucosidase, and ACE inhibition) of the olive leaf extracts. Significance levels are indicated as follows: *P < 0.05; **P < 0.01; ***P < 0.001.

The correlation between TFC, TPC, phytochemicals content, and the *in vitro* biological activities (DPPH; ABTS; FRAP; α-glucosidase, α-amylase, and ACE inhibition) was determined through Spearman’s correlation, and the results were presented with a heat map (Figure 3 and Supplementary Table 2). A highly significant positive correlation existed between TFC and DPPH (*r* = 0.9024; *P* < 0.001), ABTS (*r* = 0.7763; *P* < 0.001, and the FRAP values (*r* = 0.8069; *P* < 0.001). Among the flavonoids, significant correlations (*r* = 0.459–0.621; *P* < 0.01) were observed between the apigenin, hispidulin, luteolin, taxifolin, luteolin-7-*O*-glucoside, and luteolin-4′-*O*-glucoside and DPPH, FRAP, and ABTS values. Additionally, eriodictyol, quercetin, quercetin-3-*O*-glucoside, and kaempferol-7-*O*-glucoside showed highly significant (*P* < 0.001) positive correlations with antioxidant activity (*r* = 0.603–0.784). These results showed that flavonoids were important contributors to the antioxidant activity of the olive leaf extracts. This conclusion was consistent with the findings of a previous study by Benavente-Garcia et al. (8), which showed that flavonoids with catechol structures were the most efficient olive phenolic compound quenchers for the ABTS radical cation. In addition, the positive correlations between the triterpenic acids (e.g., maslinic, oleanonic, and oleanolic acids), chlorogenic acid, and hydroxytyrosol with antioxidant activities (ABTS, DPPH, and FRAP; *P* < 0.01) were significant. However, secoxyloganin and oleuropein showed little influence on antioxidant activity (*r* = 0.11–0.35), despite their high concentrations in the olive leaves. A previous study also reported that the oleuropein content was not well correlated with the Trolox equivalent antioxidant capacity (*r* = 0.466) because the antioxidant activity of oleuropein was mainly due to its aglycones, i.e., the hydroxytyrosol moiety in its structure (30). The glycosides in polyphenols may have reduced the radical scavenging activity, due to the diminishing coplanar B ring and the occupation of hydroxyl groups (52). Likewise, hydroxytyrosol 4-*O*-glucoside, apigenin-7-*O*-glucoside, and luteolin-7-*O*-glucoside exhibited lower correlations with antioxidant activity, compared with their corresponding aglycones.

TFC also showed significant (*r* = 0.5575, 0.6882, and 0.5644; *P* < 0.001) positive correlations with α-amylase, α-glucosidase, and ACE inhibition, and TPC exhibited a significant positive correlation (*r* = 0.5828, *P* < 0.001) with α-amylase inhibition. Apigenin, hispidulin, luteolin, taxifolin (α-glucosidase), taxifolin-3-glucoside (α-glucosidase), eriodictyol, quercetin, hydroxytyrosol, oleanolic acid (α-glucosidase), and ursolic acid (α-glucosidase) showed positive correlations with antidiabetic activity (*P* < 0.01). A previous study reported that flavonoids, such as quercetin, luteolin, and eriodictyol, could inhibit starch digestion enzymes due to their ability to non-covalently bind with the active sites of enzymes (15). According to Collado-González et al., hydroxytyrosol and oleanolic acid were possibly important contributors to the antidiabetic activity of Spanish extra virgin olive oil (53). Furthermore, apigenin, hispidulin, luteolin, eriodictyol, quercetin, taxifolin-3-glucoside, hydroxytyrosol, chlorogenic acid, and oleanolic acid exhibited statistically significant (*P* < 0.01) correlations with ACE inhibition. These results suggested that the flavonoids (e.g., quercetin, luteolin, eriodictyol, kaempferol-7-*O*-glucoside, and luteolin-7-*O*-glucoside), hydroxytyrosol, and oleanolic acid in olive leaves were correlated with the bioactive potential of olive leaf extracts.

## Conclusion

In this study, we compared the effects of freeze-drying and hot air-drying on the phytochemical profiles and biological activities of olive leaves. The drying process enhanced the release of extractable phenolics in the olive leaves. Compared to fresh olive leaves, hot air-drying effectively increased the iridoid content (i.e., oleuropein and secoxyloganin), while freeze-drying resulted in significantly higher contents of flavonoids (i.e., luteolin, quercetin, kaempferol, apigenin, hispidulin, eriodictyol, and taxifolin) and hydroxytyrosol. Among all of the treatments, FD exhibited the best radical scavenging activity and α-amylase, α-glucosidase, and ACE inhibition ability. The biological activity of the olive leaves was dependent on their phytochemical profiles. Correlation analysis indicated that the flavonoids (e.g., quercetin, luteolin, eriodictyol, kaempferol-7-*O*-glucoside, and luteolin-7-*O*-glucoside), oleanolic acid, and hydroxytyrosol were the major contributors to the biological activities of the olive leaves. This study suggested that freeze-drying was a better technique compared to hot air-drying, to enhance the flavonoid content and biological activity of dried olive leaves. In addition, hot air-drying was a viable alternative drying method to ensure the maximal recovery of iridoids.

## Data Availability Statement

The original contributions presented in the study are included in the article/Supplementary Material, further inquiries can be directed to the corresponding author/s.

## Author Contributions

CZ: conceptualization, methodology, and writing—original draft. JZ: software and writing—review and editing. XX: methodology and data curation. SZ: funding acquisition and resources. EN: resources. QW and TL: methodology and data curation. DL: supervision and writing—review and editing. All authors contributed to the article and approved the submitted version.

## Conflict of Interest

The authors declare that the research was conducted in the absence of any commercial or financial relationships that could be construed as a potential conflict of interest.

## Publisher’s Note

All claims expressed in this article are solely those of the authors and do not necessarily represent those of their affiliated organizations, or those of the publisher, the editors and the reviewers. Any product that may be evaluated in this article, or claim that may be made by its manufacturer, is not guaranteed or endorsed by the publisher.
