# Supplementary material for: Changes in Phytochemical Profiles and Biological Activity of Olive Leaves Treated by Two Drying Methods
Source: Front Nutr. 2022 Apr 28;9:854680. doi: 10.3389/fnut.2022.854680 (PMC9097227; doi:10.3389/fnut.2022.854680)
Supplement: Supplementary file 1 [file Data_Sheet_1.doc]

# Changes in phytochemical profiles and biological activity of olive leaves treated by two drying methods

Chengcheng Zhang^1^, Jianming Zhang^1^, Xiaoting Xin^1^, Shenlong Zhu^2^, Erli Niu^2^, Qinghang Wu^1^, Ting Li^1^, Daqun Liu^1,*^

1 Food Science Institute, Zhejiang Academy of Agricultural Sciences, Hangzhou 310021, China

2 Institute of Crop and Nuclear Technology Utilization, Zhejiang Academy of Agricultural Sciences, Hangzhou 310021, China

*Corresponding author:

Daqun Liu, E-mail: [liudaqun@zaas.ac.cn](mailto:liudaqun@zaas.ac.cn)

Table S1. Calibration curves used for HPLC quantification.

| Compounds | Rt (min) | Calibration curves | R^2^ | Linear range  (μg/mL) |
| --- | --- | --- | --- | --- |
| [Hydroxytyrosol](https://www.chemsrc.com/en/cas/10597-60-1_522847.html) | 5.337 | Y = -11733+9341.9*X | 0.9999 | 13.75-275 |
| Chlorogenic acid | 7.070 | Y =-3292+6252.1*X | 0.9998 | 4.6-91.7 |
| Secoxyloganin | 8.430 | Y =1395+4572.1*X | 1.0000 | 23.1-461.5 |
| Plantamajoside | 9.550 | Y =-1654+2404.5*X | 0.9995 | 13.8-276.9 |
| Rutin | 11.671 | Y =-432+6939.3*X | 0.9994 | 4.8-96.7 |
| Luteolin-7-O-glucoside | 12.597 | Y =3006+7658.7*X | 0.9994 | 5.1-102.8 |
| Apigenin-7-O-neohesperidoside | 13.803 | Y =3426+5625.8*X | 0.9983 | 6.7-133.4 |
| Taxifolin | 14.055 | Y = -1568+16950.9*X | 0.9998 | 18.5-369.2 |
| Quercetin | 14.35 | Y = -11817+12587.5*X | 0.9999 | 8.8-175.6 |
| Oleuropein | 16.419 | Y =1459+5382.6*X | 0.9998 | 25.6-513.3 |
| Eriodictyol | 20.234 | Y = -1236+6175.2*X | 1.0000 | 28.8-576.8 |
| Luteolin | 21.134 | Y = -5079+19174.8*X | 0.9995 | 2.2-43.3 |
| Kaempferol | 24.857 | Y = -13367+18813.5*X | 1.0000 | 5.8-116.6 |
| Apigenin | 26.870 | Y = -12297+17367.4*X | 0.9999 | 5.3-100.5 |
| Asiatic acid | 3.554 | Y =2560.5+ 4126.9*X | 0.9999 | 3.24-323 |
| Oleanonic acid | 4.725 | Y = -1451.8+4198.4*X | 0.9996 | 3.7-369 |
| Maslinic acid | 5.865 | Y = -4574.9+3976.8*X | 0.9999 | 3.24-323 |
| Corosolic acid | 6.187 | Y =779.67+4236.5*X | 1.0000 | 3.92-392 |
| Oleanolic acid | 9.927 | Y=1613.1+5762.8*X | 0.9999 | 2.3-230 |
| Ursolic acid | 10.232 | Y= 8616.1+4334.9*X | 1.0000 | 3.46-346 |

Rt, retention times; R^2^, correlation coefficients.

Table S2. Spearman’s correlation co-efficient analysis between chemical constituents and the bioactivities (DPPH; ABTS; FRAP; α-amylase, α-glucosidase, and ACE inhibition) of olive leaf extracts.

| Analytes | DPPH | FRAP | ABTS | α-amylase | α- Glucosidase | ACE |
| --- | --- | --- | --- | --- | --- | --- |
| Apigenin | 0.4595 | 0.4652 | 0.4966 | 0.7711 | 0.3283 | 0.4889 |
| Apigenin-7-O-glucoside | 0.0532 | -0.0290 | -0.0458 | -0.2618 | -0.3447 | -0.3879 |
| Apigenin-7-O-neohesperidoside | -0.2146 | -0.2167 | -0.0602 | -0.2118 | 0.0795 | -0.1876 |
| Asiatic acid | 0.0316 | 0.0303 | 0.0756 | 0.0371 | 0.0422 | -0.0566 |
| Chlorogenic acid | 0.5866 | 0.5430 | 0.5040 | 0.3509 | 0.3927 | 0.6157 |
| Corosolic acid | 0.2195 | 0.1953 | 0.1953 | 0.4644 | 0.2371 | 0.3040 |
| Coumarin | 0.3706 | 0.2805 | 0.2373 | -0.1910 | -0.0893 | 0.0754 |
| Diosmetin-7-O-neohesperidoside | 0.6036 | 0.4888 | 0.4859 | 0.2154 | 0.4533 | 0.4528 |
| Eriodictyol | 0.7745 | 0.6635 | 0.6602 | 0.5315 | 0.5109 | 0.6466 |
| Esculin | 0.5824 | 0.5333 | 0.4779 | 0.2203 | 0.0247 | 0.3326 |
| Hispidulin | 0.5137 | 0.4872 | 0.5850 | 0.8152 | 0.7477 | 0.6260 |
| Hydroxytyrosol | 0.8486 | 0.8051 | 0.7418 | 0.4556 | 0.4855 | 0.6237 |
| Hydroxytyrosol 4-O-glucoside | 0.2095 | 0.1738 | 0.1189 | -0.1741 | 0.2302 | 0.1968 |
| Kaempferol | 0.0994 | 0.0407 | 0.0789 | 0.3399 | 0.3758 | 0.1433 |
| Kaempferol-7-O-glucoside | 0.7323 | 0.6298 | 0.6033 | 0.3099 | 0.2515 | 0.5390 |
| Luteolin | 0.5142 | 0.5191 | 0.5631 | 0.7977 | 0.6301 | 0.5848 |
| Luteolin-3',7-di-O-glucoside | 0.5659 | 0.4562 | 0.4905 | 0.1426 | 0.3612 | 0.3981 |
| Luteolin-4'-O-glucoside | 0.6126 | 0.5181 | 0.4718 | 0.1580 | 0.3902 | 0.5202 |
| Luteolin-7-O-glucoside | 0.6205 | 0.5245 | 0.4594 | 0.1910 | 0.0762 | 0.3459 |
| Maslinic acid | 0.5513 | 0.5698 | 0.4548 | 0.0129 | 0.0981 | 0.2731 |
| Oleanolic acid | 0.6141 | 0.5871 | 0.6537 | 0.3091 | 0.6512 | 0.6170 |
| Oleanonic acid | 0.5498 | 0.5325 | 0.4473 | 0.1995 | 0.0880 | 0.2062 |
| Oleuropein | 0.3570 | 0.2692 | 0.1240 | -0.2940 | -0.2296 | -0.0299 |
| Plantamajoside | -0.2539 | -0.0880 | -0.0187 | 0.0923 | 0.0424 | 0.0456 |
| Quercetin | 0.7842 | 0.6742 | 0.6650 | 0.4355 | 0.4962 | 0.5647 |
| Quercetin-3-O-glucoside | 0.6586 | 0.6669 | 0.6329 | 0.4342 | 0.1526 | 0.4710 |
| Quercetin-4'-O-glucoside | 0.1674 | 0.1028 | 0.1689 | 0.3436 | 0.3222 | 0.1842 |
| Rutin | 0.4108 | 0.2808 | 0.2715 | 0.1125 | 0.3133 | 0.1689 |
| Secoxyloganin | 0.3137 | 0.2236 | 0.1148 | -0.3784 | -0.1470 | -0.0082 |
| Taxifolin | 0.4599 | 0.4960 | 0.5343 | 0.4216 | 0.5405 | 0.5184 |
| Taxifolin-3-glucoside | 0.4839 | 0.4169 | 0.4779 | 0.0847 | 0.4847 | 0.4880 |
| TFC | 0.9024 | 0.8069 | 0.7763 | 0.5575 | 0.5645 | 0.6883 |
| Tiliroside | 0.4128 | 0.2998 | 0.1377 | 0.2443 | 0.0427 | 0.0667 |
| TPC | 0.7706 | 0.6267 | 0.5534 | 0.5828 | 0.3400 | 0.4978 |
| Ursolic acid | 0.2630 | 0.2000 | 0.3021 | 0.4005 | 0.4680 | 0.3076 |


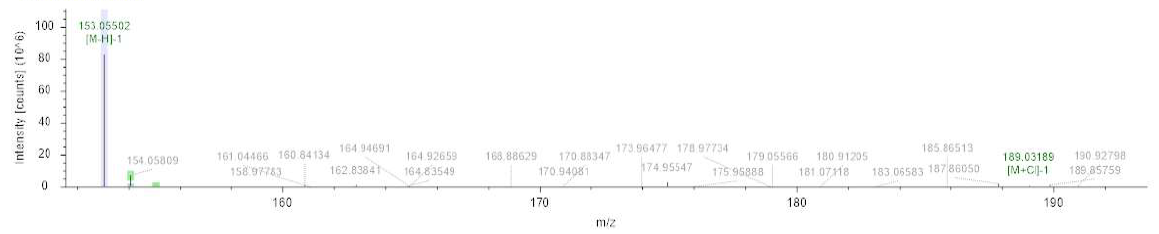

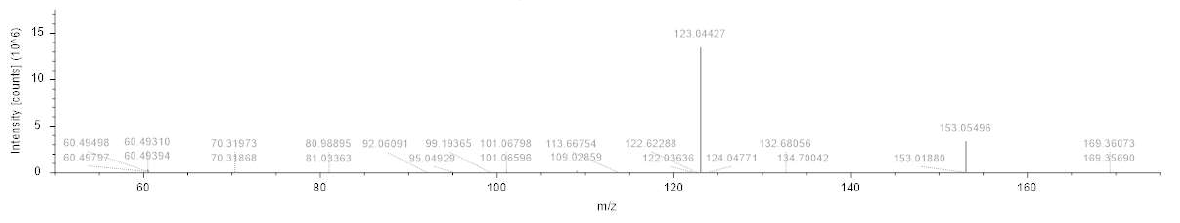


**1.** [**Hydroxytyrosol**](https://www.chemsrc.com/en/cas/10597-60-1_522847.html)


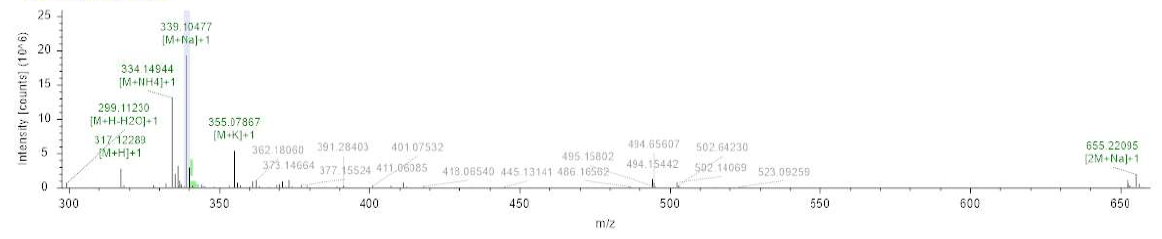


**2.** [**Hydroxytyrosol 4-O-glucoside**](https://www.chemsrc.com/en/cas/54695-80-6_327046.html)


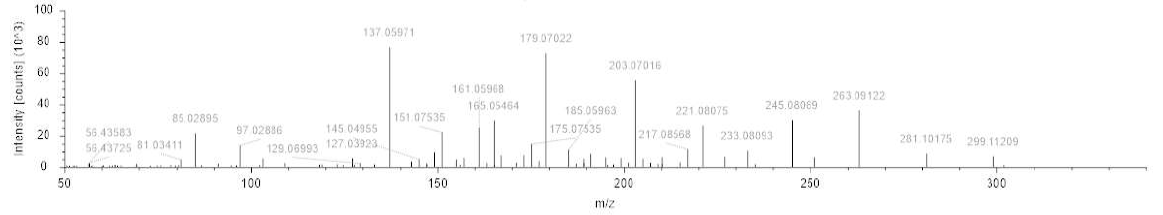


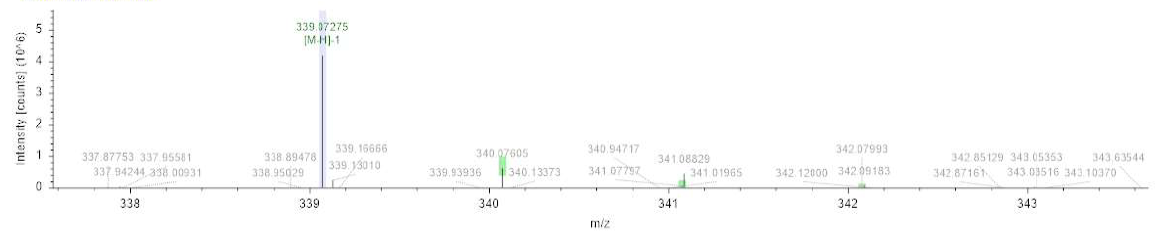


**3. Esculin**


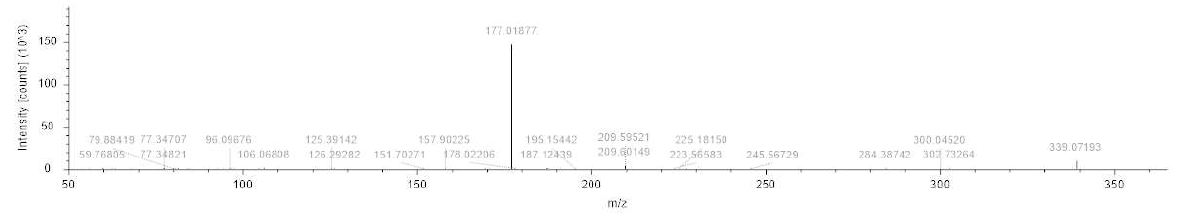


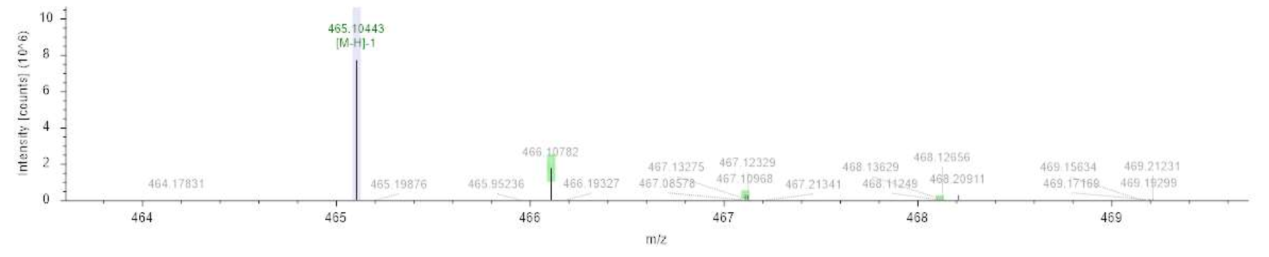

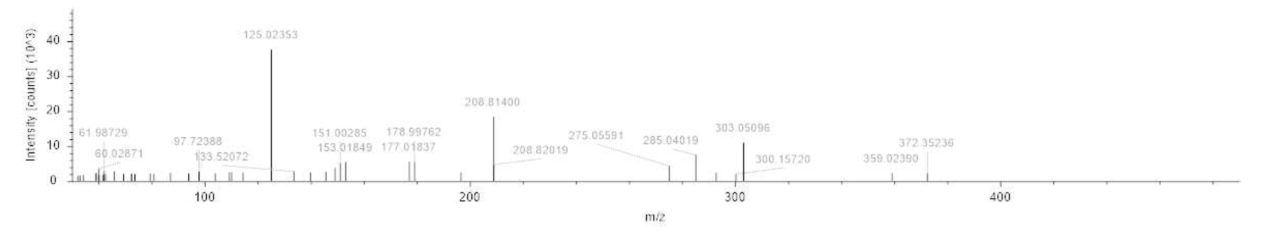


**4. Taxifolin-3-glucoside**


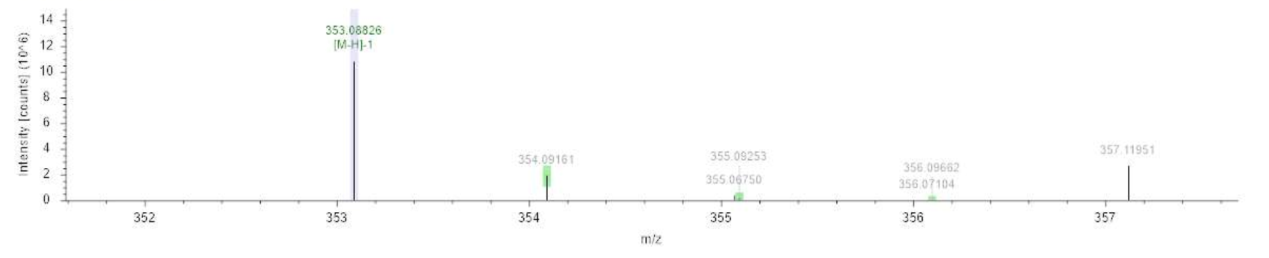

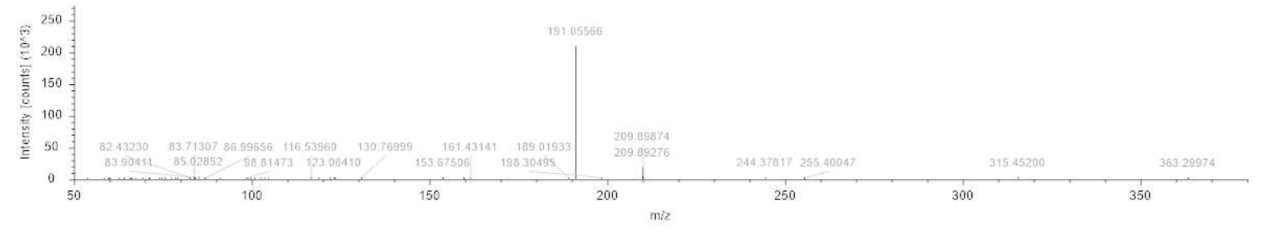


**5. Chlorogenic acid**


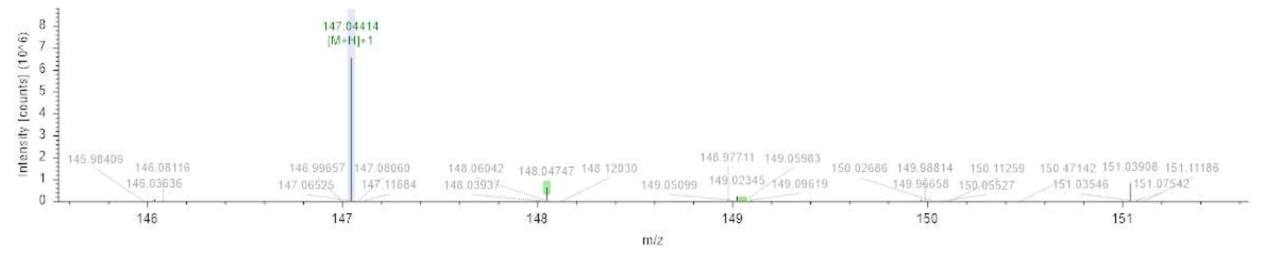


**6. Coumarin**


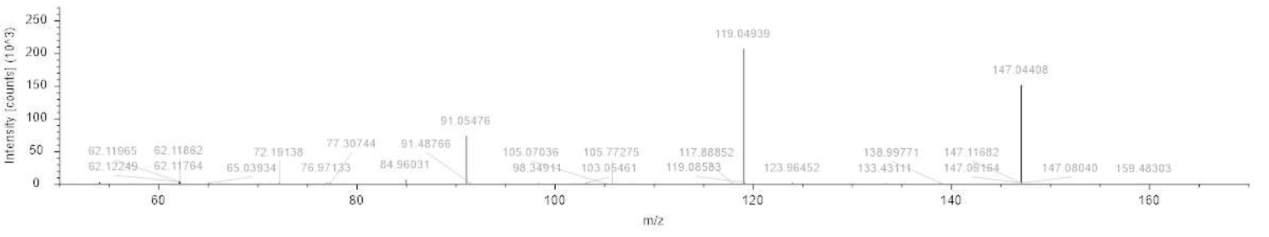


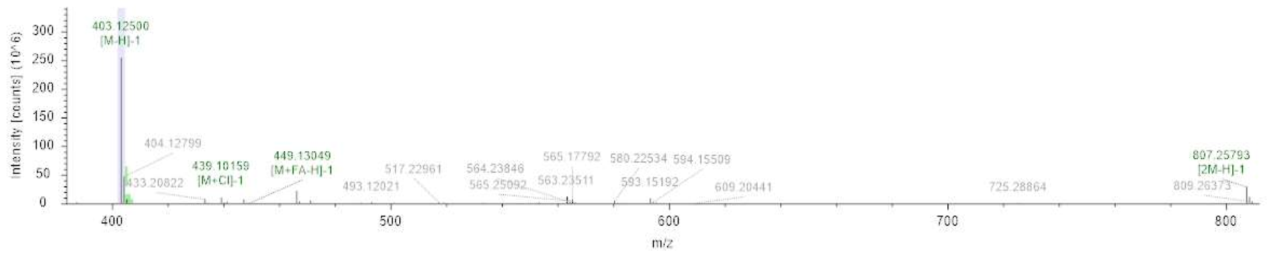


**7. Secoxyloganin**


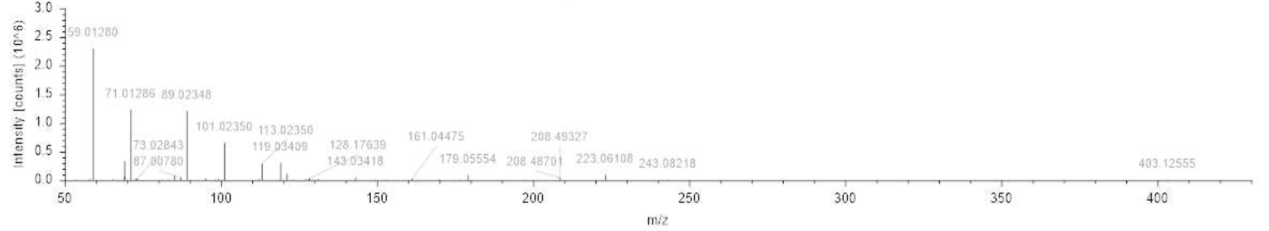


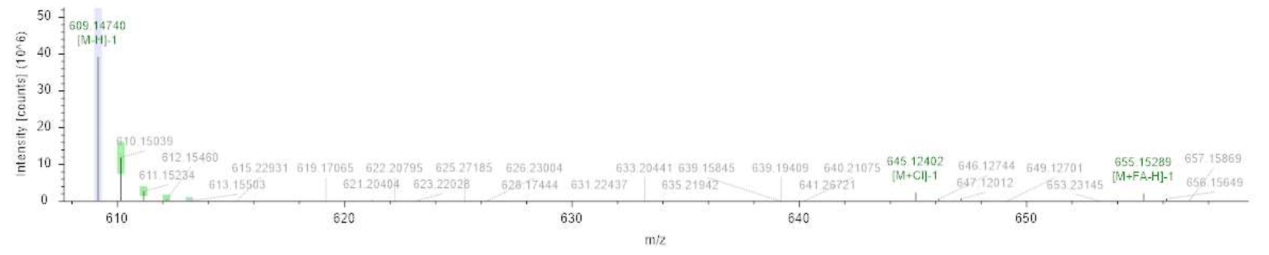


**8. Luteolin-3',7-di-O-glucoside**


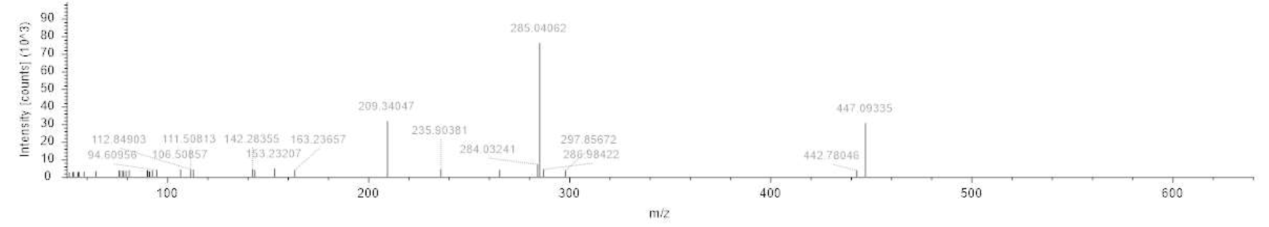


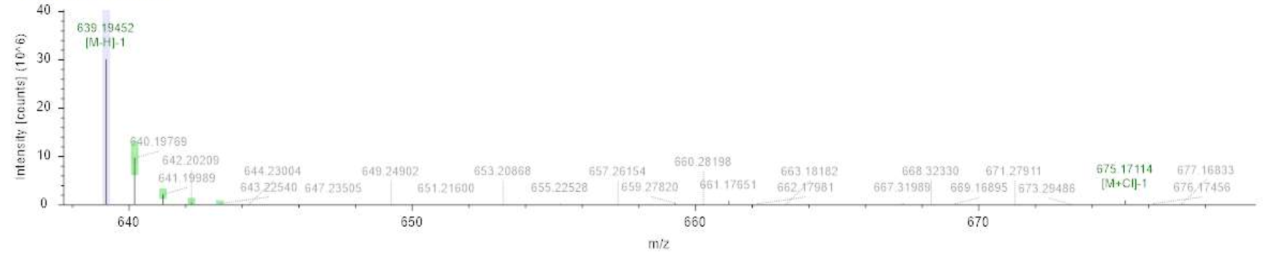


**9. Plantamajoside**


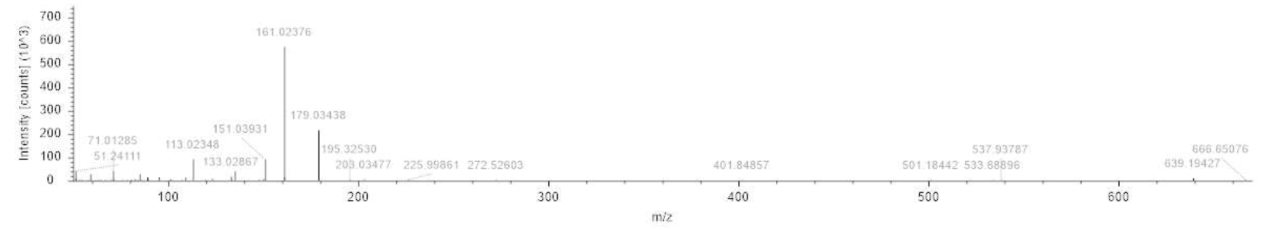


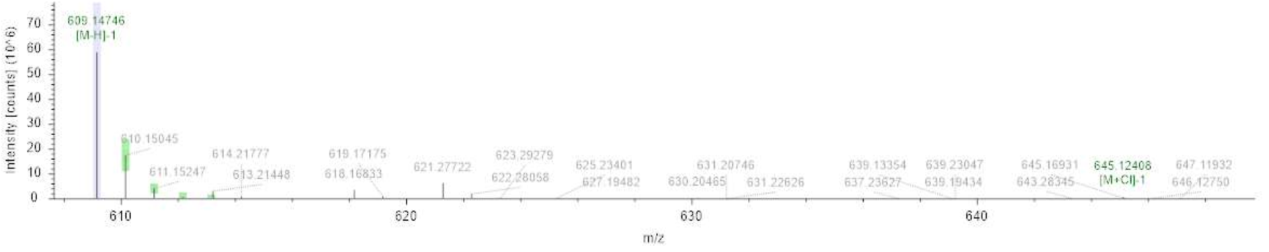


**10. Rutin**


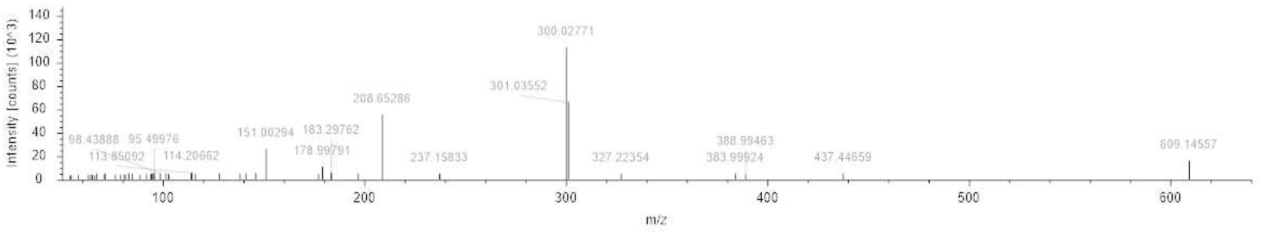


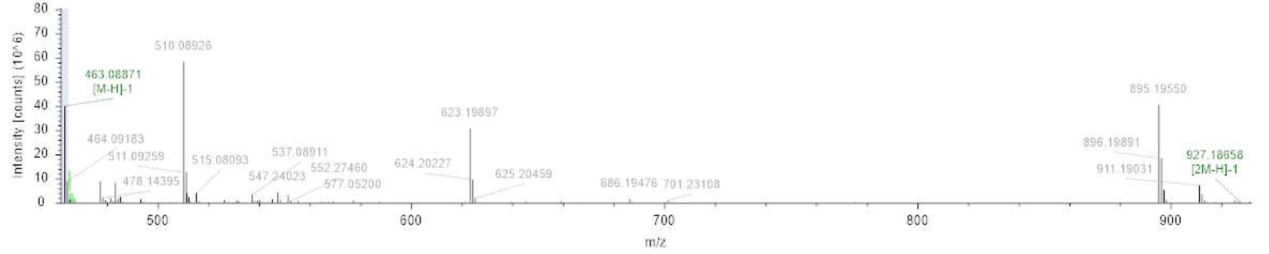


**11. Quercetin-3-O-glucoside**


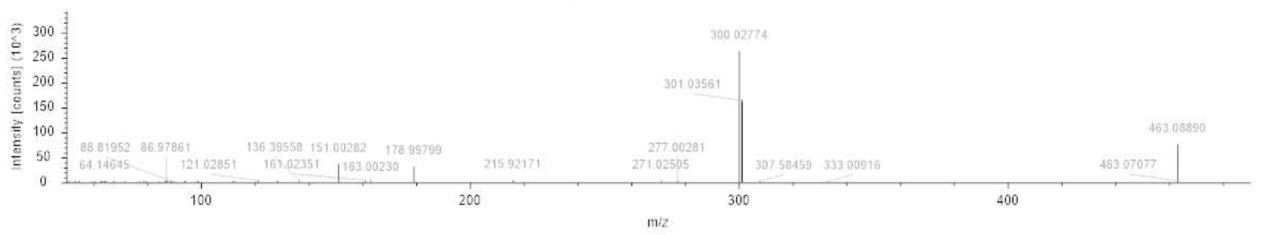


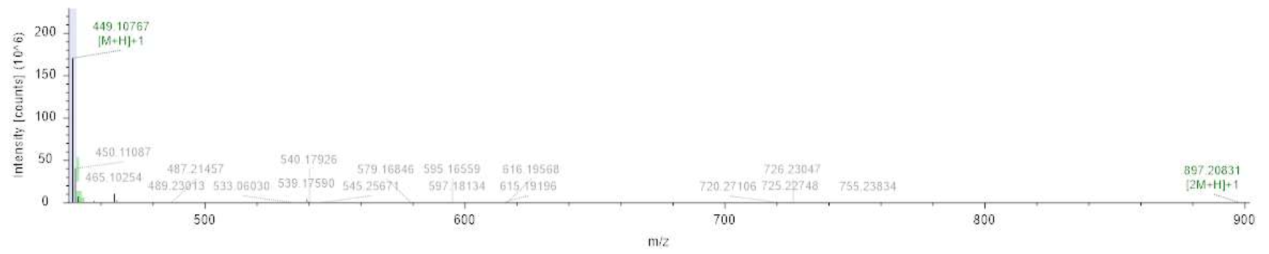

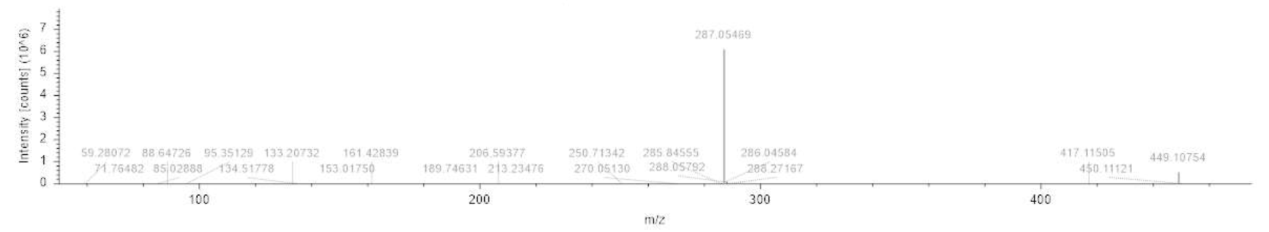


**12. Luteolin-7-O-glucoside**


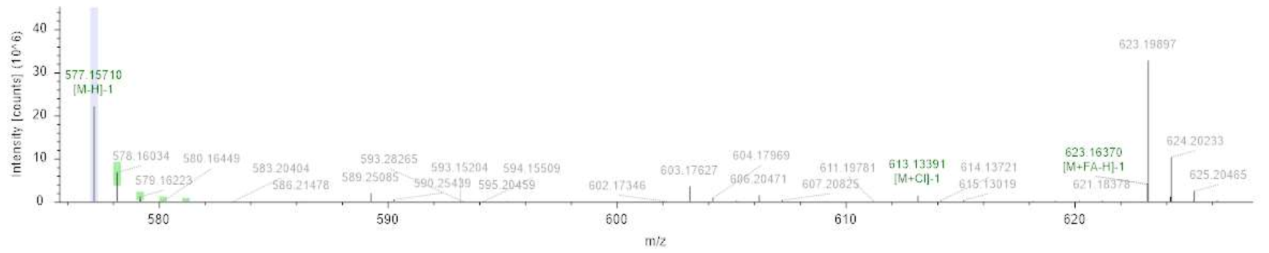


**13. Apigenin-7-O-neohesperidoside**


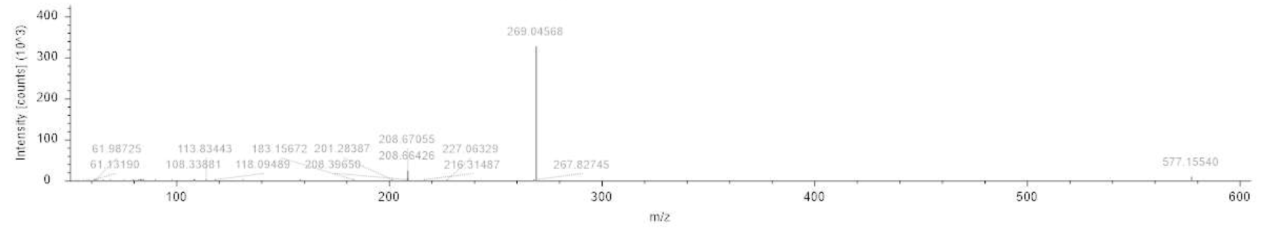


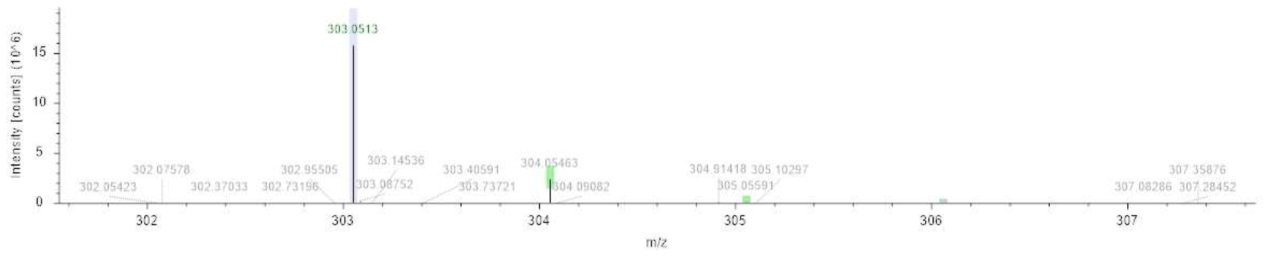


**14. Taxifolin**


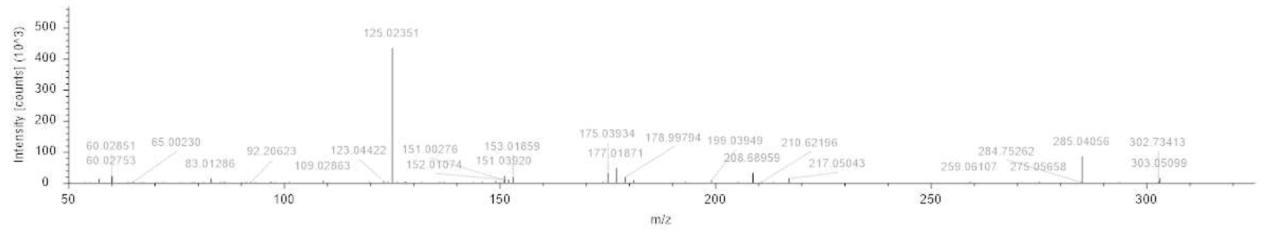


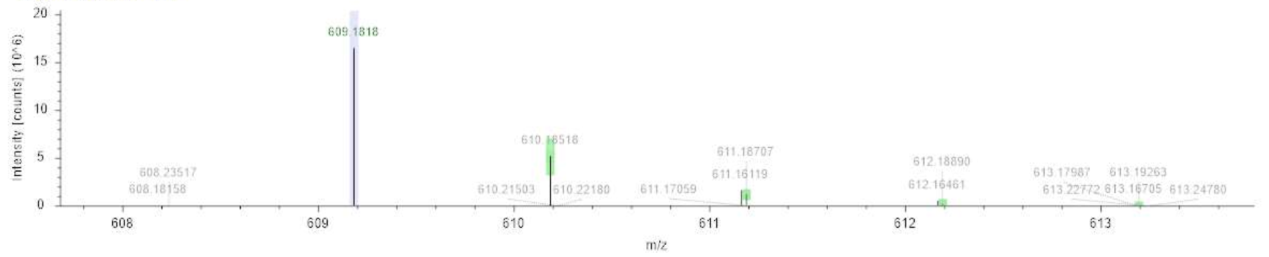


**15. Diosmetin-7-O-neohesperidoside**


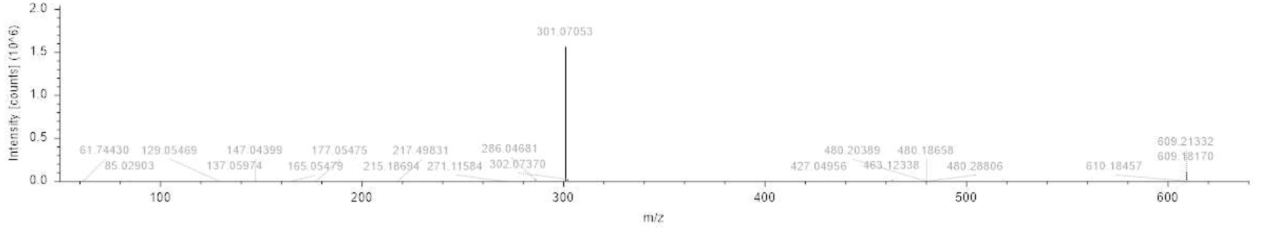


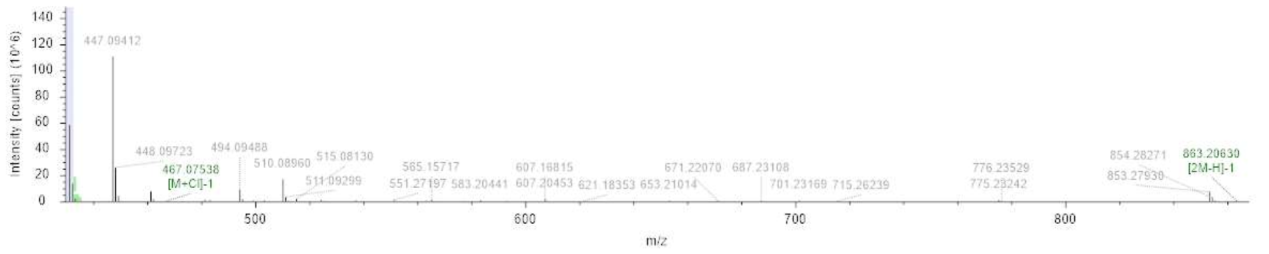


**16. Apigenin-7-O-glucoside**


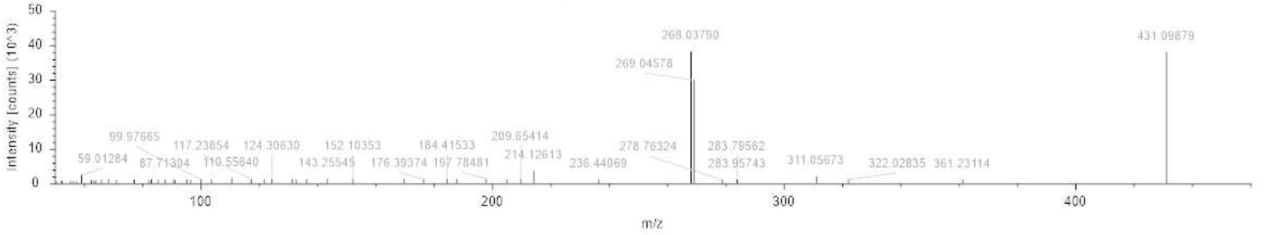


**17. Kaempferol-7-O-glucoside**


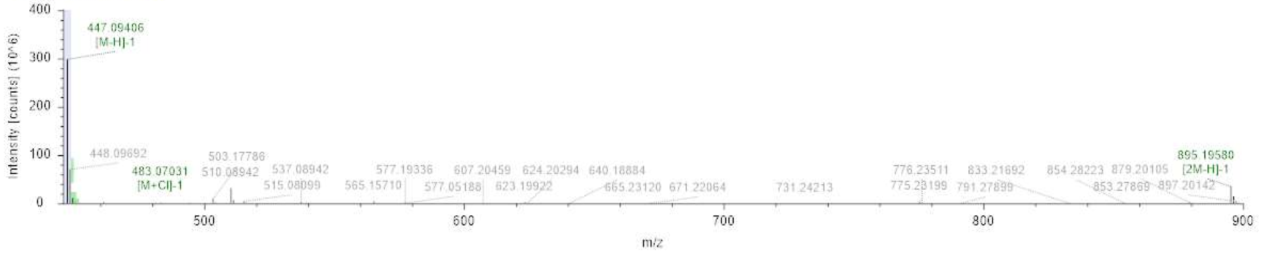


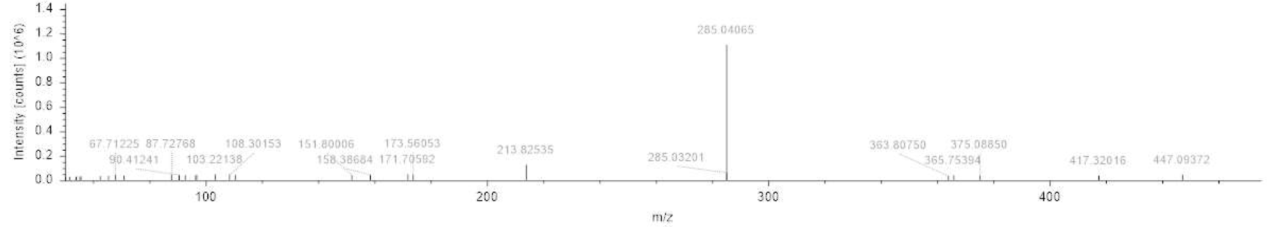


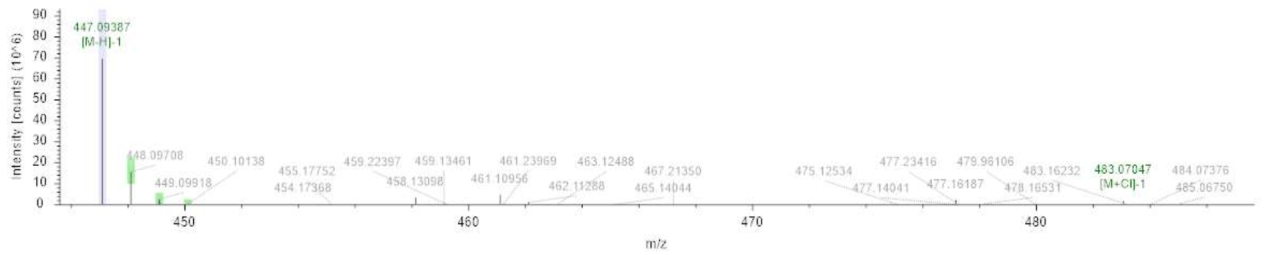


**18. Luteolin 4'-O-glucoside**


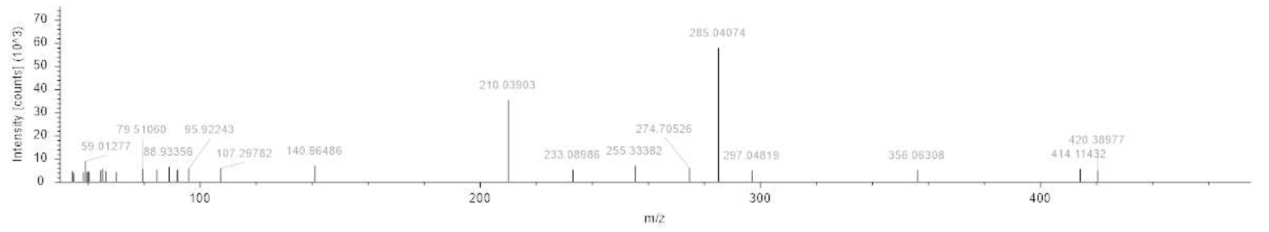


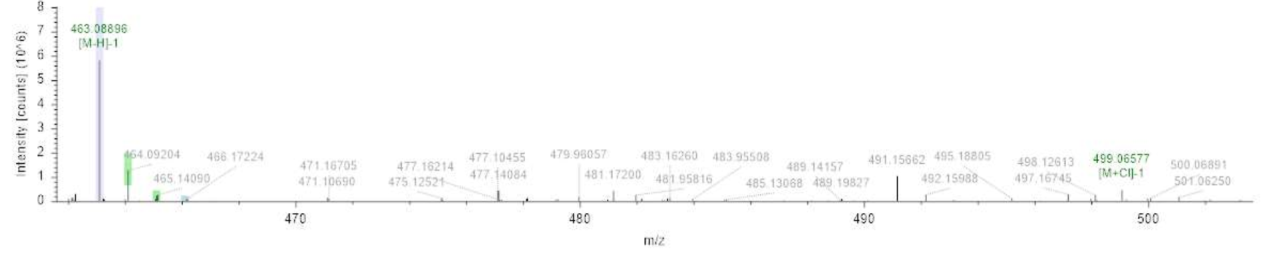


**19. Quercetin-4'-O-glucoside**


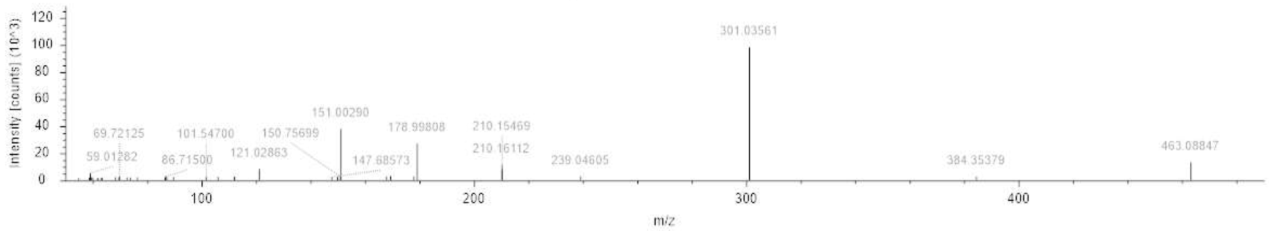


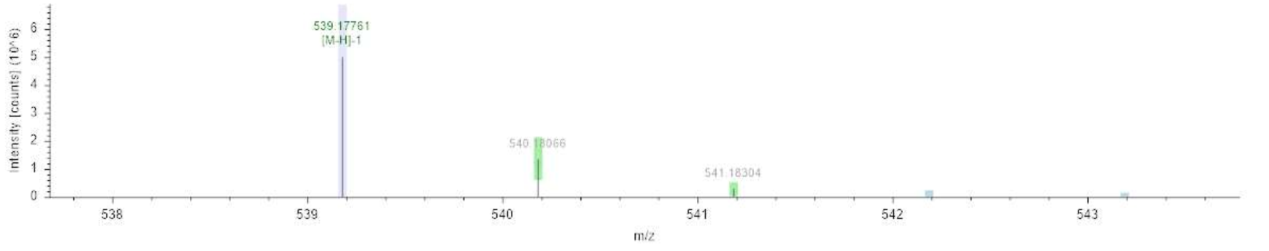


**20. Oleuropein**


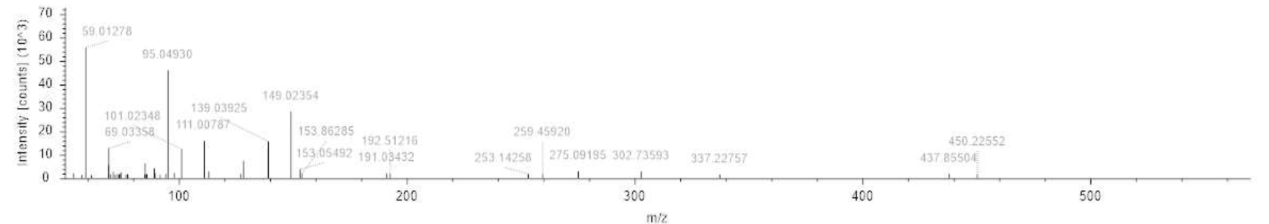


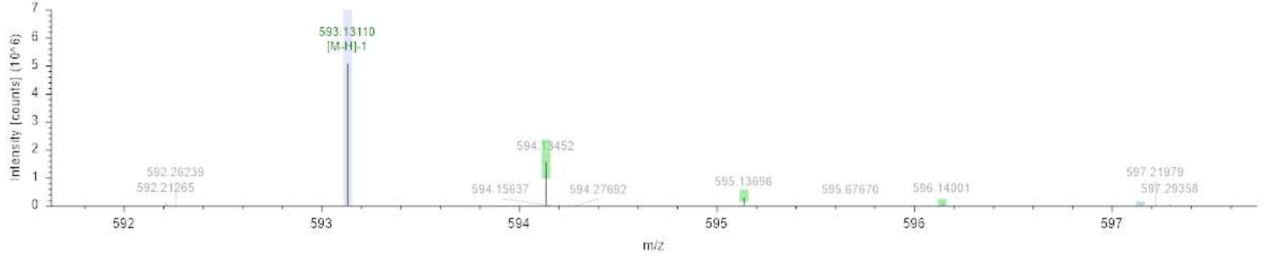


**21. Tiliroside**


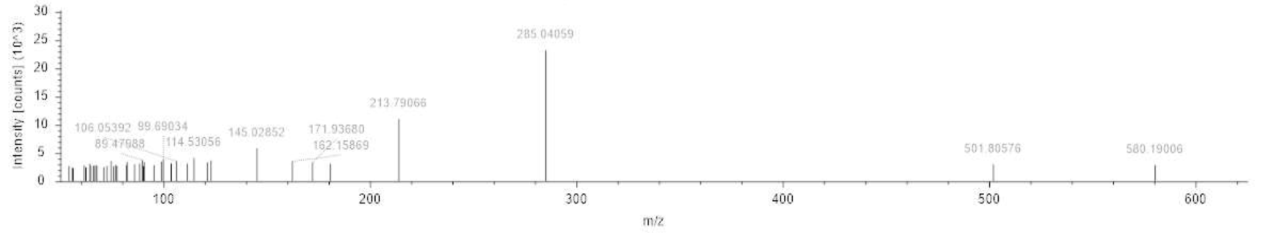


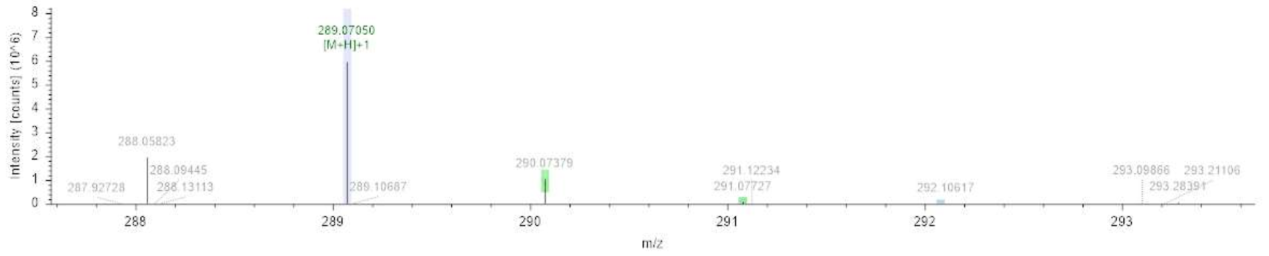


**22. Eriodictyol**


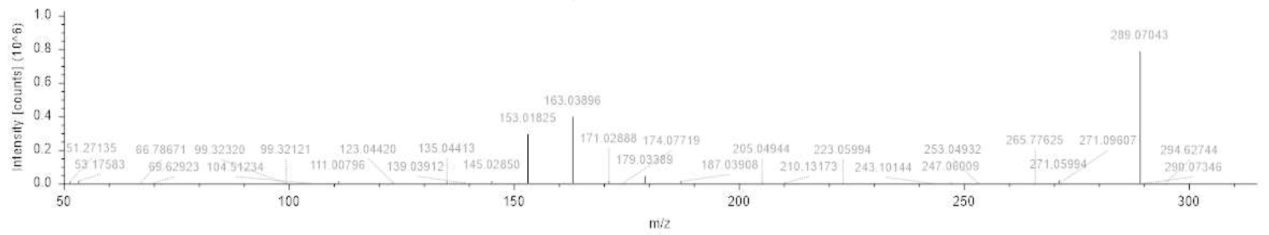


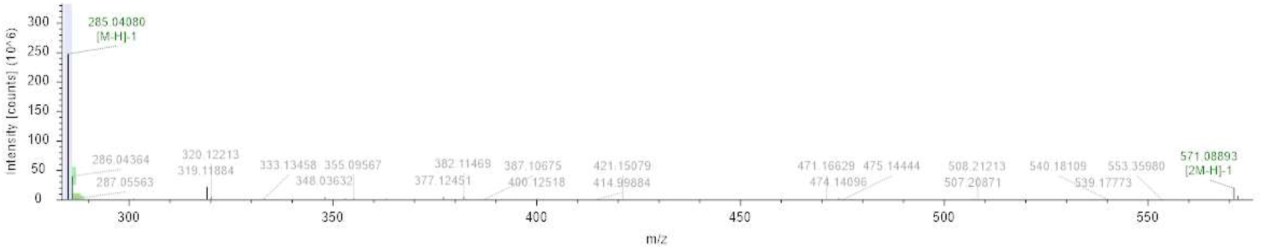


**23. Luteolin**


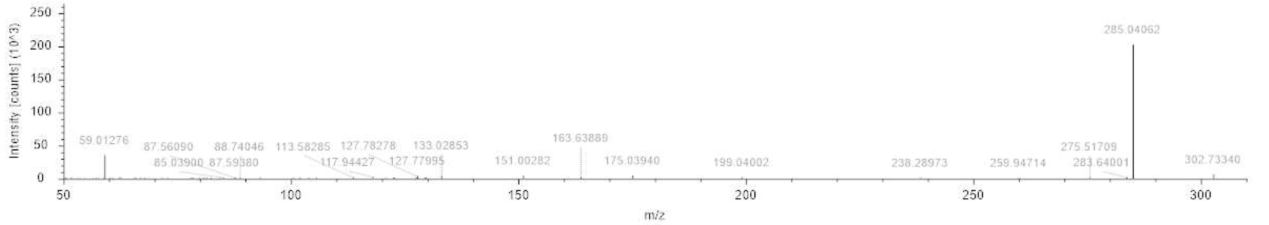


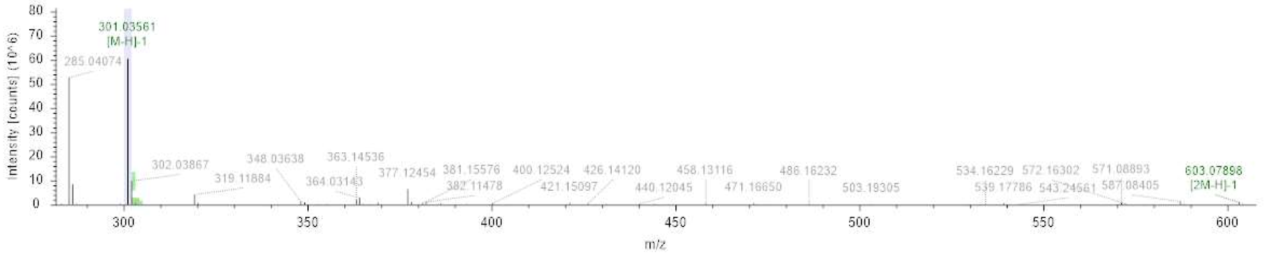


**24. Quercetin**


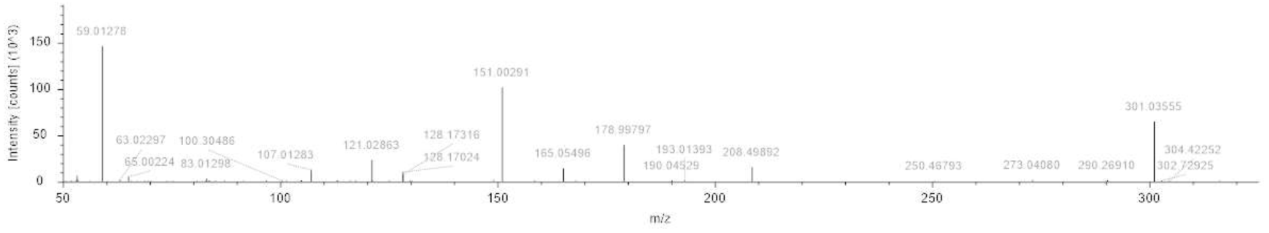


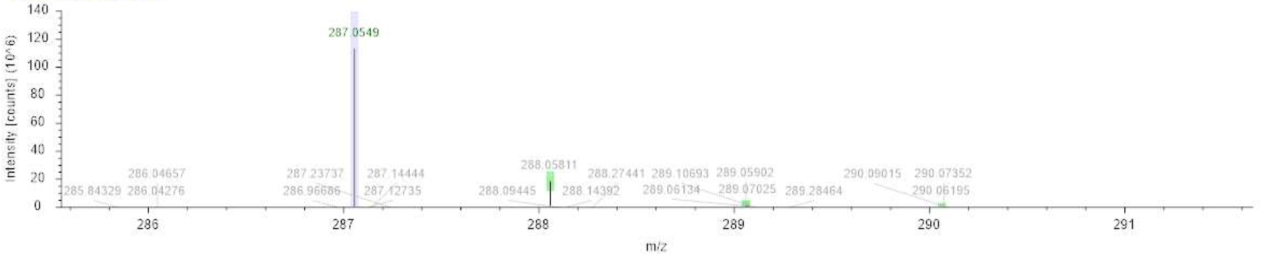


**25. Kaempferol**


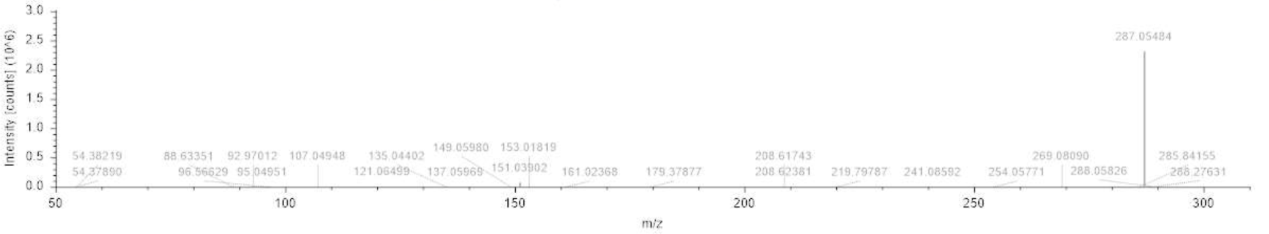


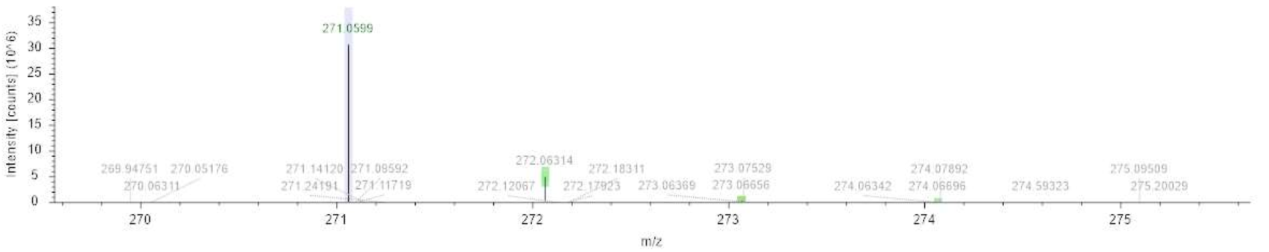


**26. Apigenin**


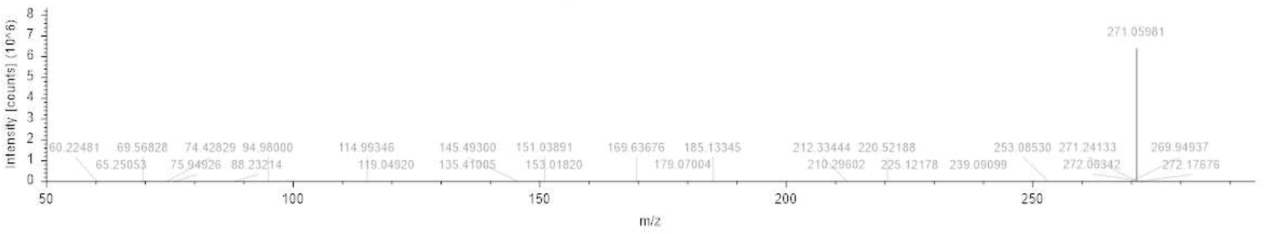


**27. Hispidulin**


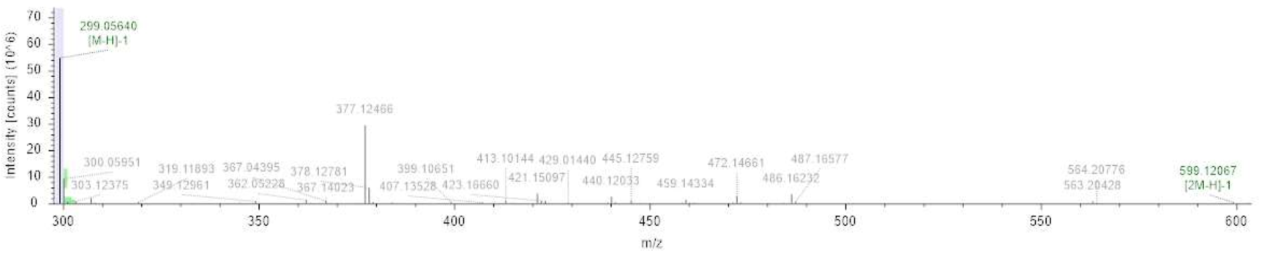


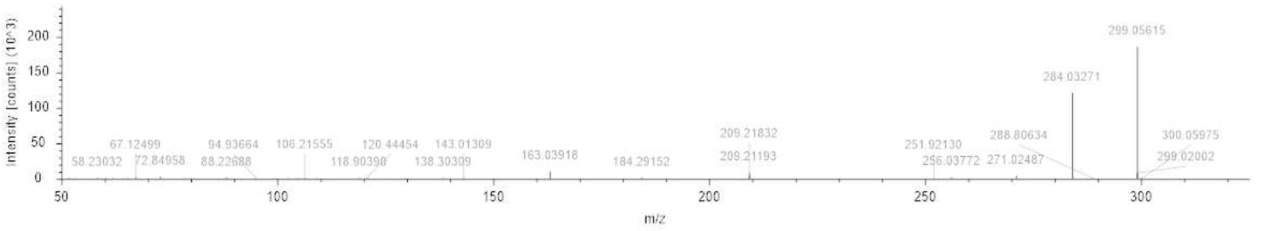


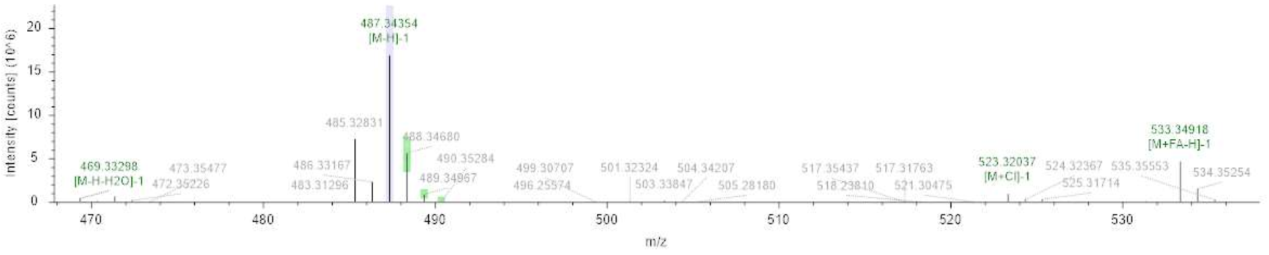


**28. Asiatic acid**


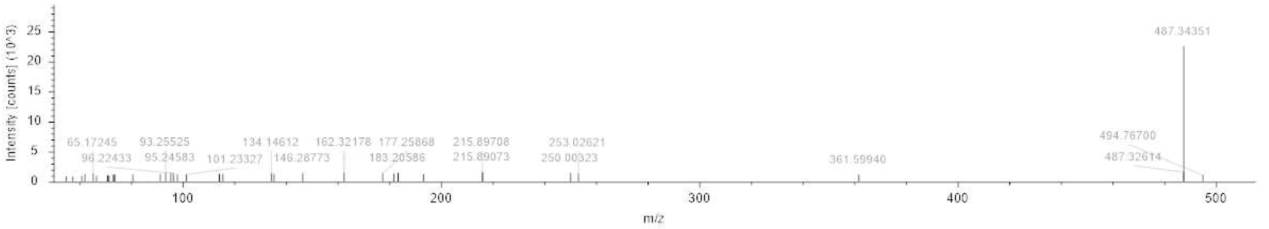


**29. Oleanonic acid**


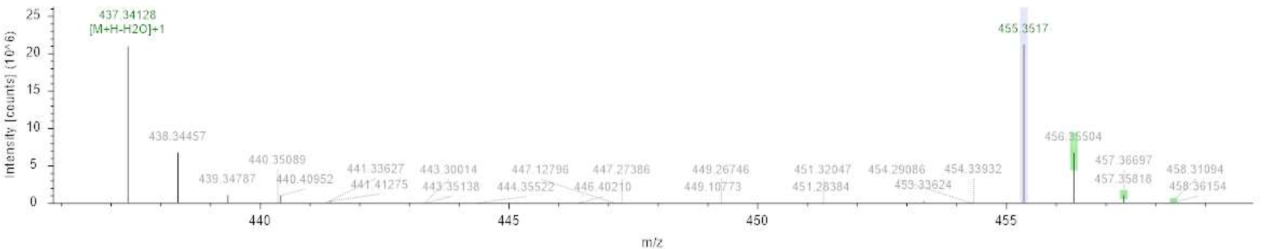


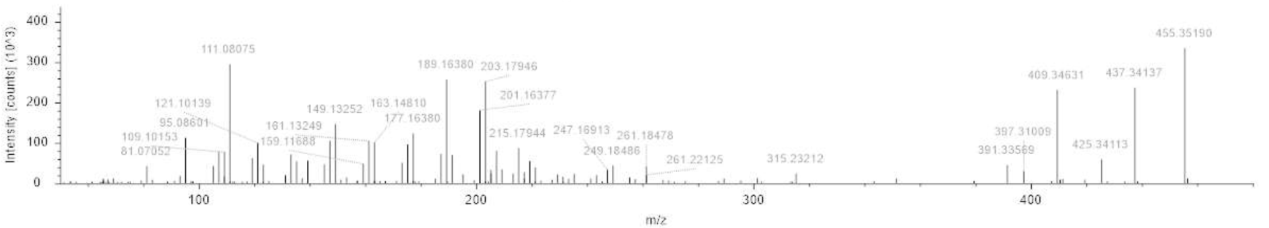


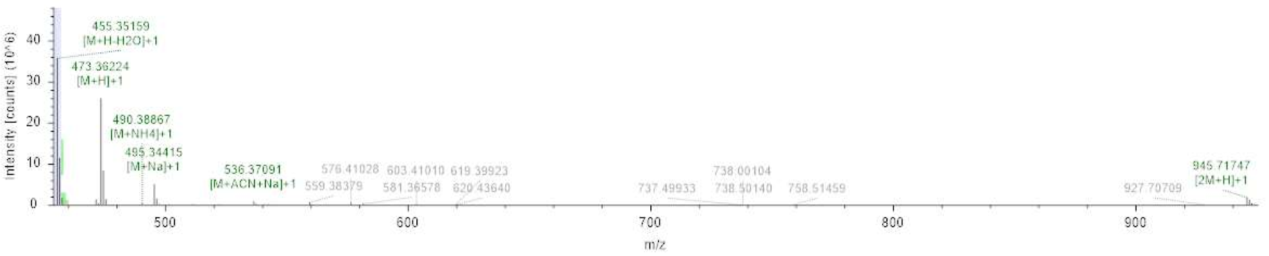


**30. Maslinic acid**


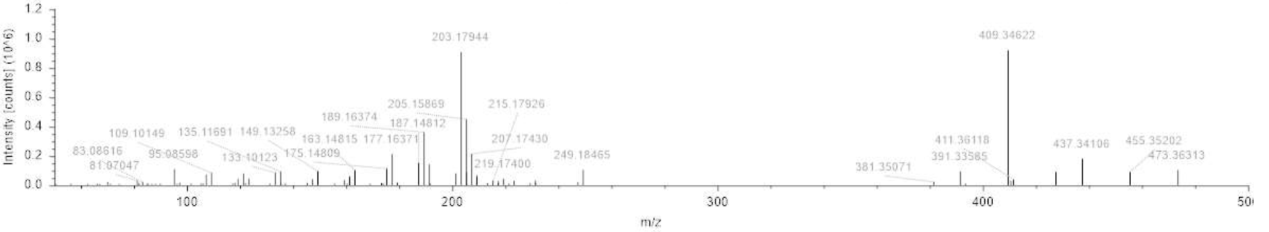


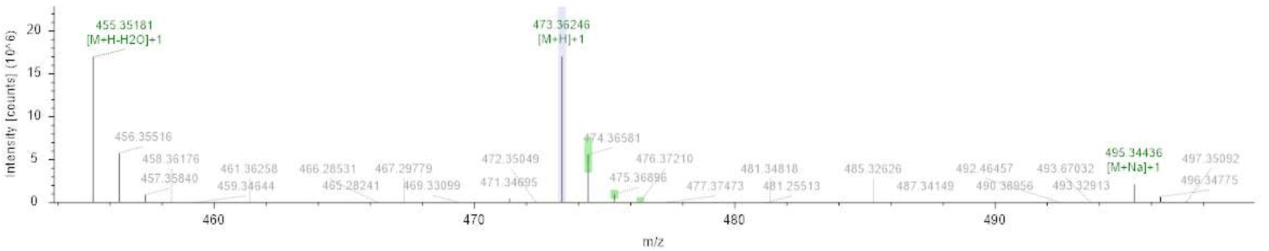


**31. Corosolic acid**


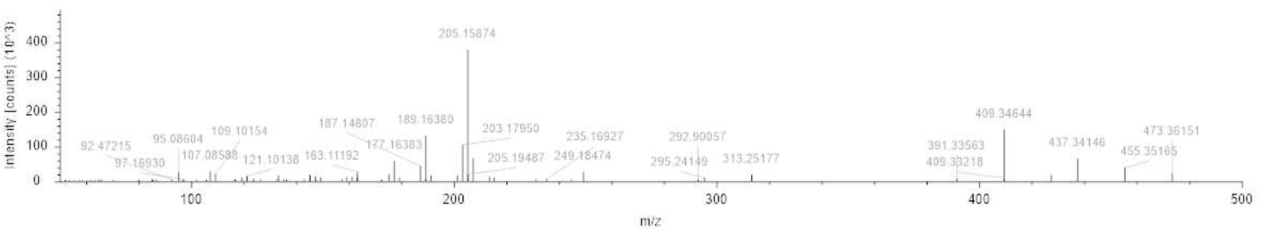


**32. Ursolic acid**


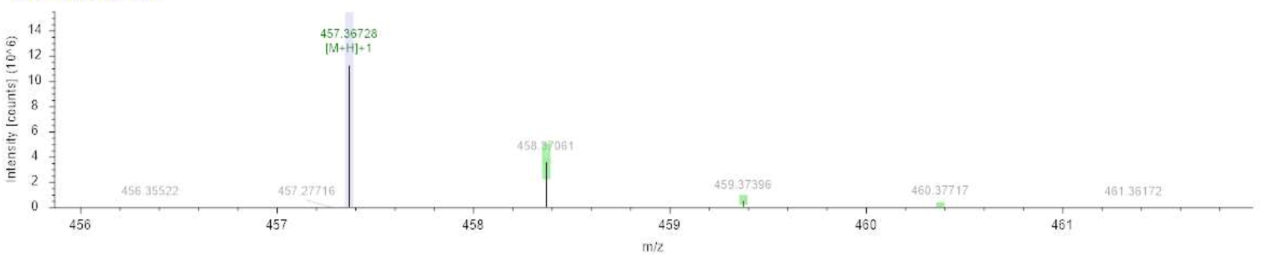


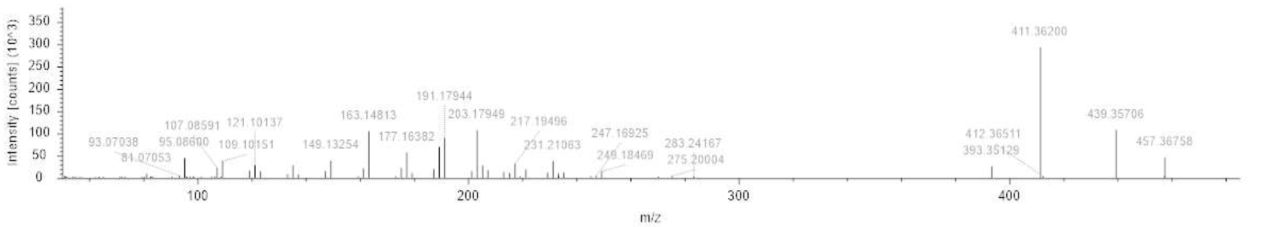


**33. Oleanolic acid**


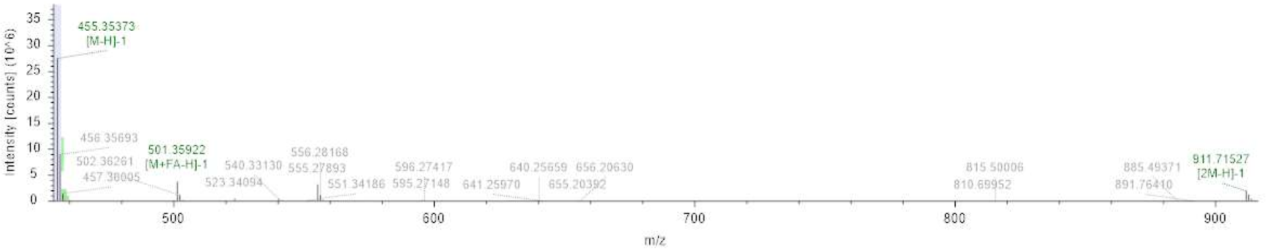


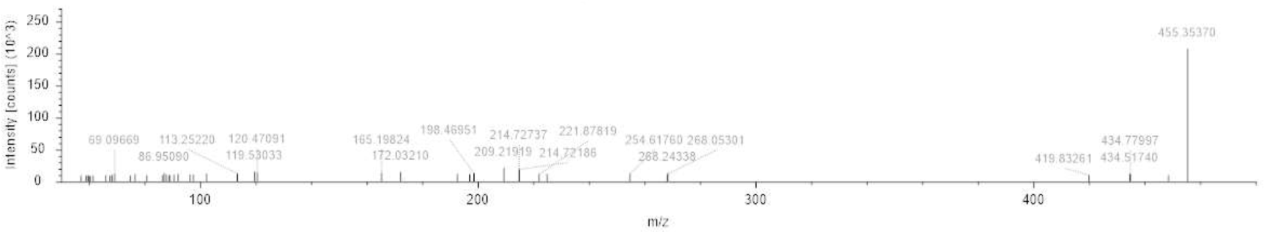


Figure S1. MS and MS/MS data of the 33 phytochemicals identified in the olive leaves by UPLC-Q-Exactive Orbitrap-MS.
